# Polypharmacy through Phage Display: Selection of Glucagon and GLP-1 Receptor Co-agonists from a Phage-Displayed Peptide Library

**DOI:** 10.1038/s41598-017-18494-5

**Published:** 2018-01-12

**Authors:** Anna Demartis, Armin Lahm, Licia Tomei, Elisa Beghetto, Valentina Di Biasio, Federica Orvieto, Francesco Frattolillo, Paul E. Carrington, Sheena Mumick, Brian Hawes, Elisabetta Bianchi, Anandan Palani, Antonello Pessi

**Affiliations:** 1 IRBM Science Park, Via Pontina Km 30.600, 00071 Pomezia, RM Italy; 2 Merck Research Laboratories, 2015 Galloping Hill R., Kenilworth, NJ 07033-1310 USA; 3 PeptiPharma, Viale Città D’Europa 679, 00144 Roma, Italy

## Abstract

A promising emerging area for the treatment of obesity and diabetes is combinatorial hormone therapy, where single-molecule peptides are rationally designed to integrate the complementary actions of multiple endogenous metabolically-related hormones. We describe here a proof-of-concept study on developing unimolecular polypharmacy agents through the use of selection methods based on phage-displayed peptide libraries (PDL). Co-agonists of the glucagon (GCG) and GLP-1 receptors were identified from a PDL sequentially selected on GCGR- and GLP1R-overexpressing cells. After two or three rounds of selection, 7.5% of randomly picked clones were GLP1R/GCGR co-agonists, and a further 1.53% were agonists of a single receptor. The phages were sequenced and 35 corresponding peptides were synthesized. 18 peptides were potent co-agonists, 8 of whom showed EC_50_ ≤ 30 pM on each receptor, comparable to the best rationally designed co-agonists reported in the literature. Based on literature examples, two sequences were engineered to stabilize against dipeptidyl peptidase IV cleavage and prolong the *in vivo* half-life: the engineered peptides were comparably potent to the parent peptides on both receptors, highlighting the potential use of phage-derived peptides as therapeutic agents. The strategy described here appears of general value for the discovery of optimized polypharmacology paradigms across several metabolically-related hormones.

## Introduction

One of the most promising emerging areas for the treatment of obesity and Diabetes is combinatorial hormone therapies^1–4^. In particular, single molecule peptides have been discovered integrating the complementary actions of multiple endogenous metabolically-related hormones^5–18^.

One particularly interesting combination is found in peptides that simultaneously activate the glucagon receptor (GCGR) and the glucagon-like peptide-1 receptor (GLP1R)^8^. Glucagon has anti-obesity activity by reducing food intake^19,20^, and inducing thermogenesis and stimulation of growth of the brown adipose-tissue^21^, but raises blood glucose by stimulating gluconeogenesis and glycogenolysis. The latter effect can be counteracted by the antihyperglycemic property of GLP-1, which enhances glucose-stimulated insulin synthesis and secretion^22^.

Careful optimization of the relative potency at each receptor for a series of GCGR/GLP1R co-agonists^11^ led to potent antidiabetic and antiobesity peptides^8^.

The high evolutionary relatedness of peptides like glucagon and GLP-1, enabled using the sequence of one of these hormones as a starting point, onto which co-agonism for the other receptor could be installed, with relatively few sequence changes^8,9,11^. Sometimes, as little as one amino acid change is sufficient to switch between single- and dual-hormone agonism^10,23^.

These studies have been carried out by “rational design”, through an iterative cycle combining hybridization of the parent peptides with single-point mutations guided by the developing SAR: while successful and a testimony to the ingenuity of peptide medicinal chemists, this has necessarily limited the exploration of the chemical space to the immediate vicinity of the parent sequences. For example, the effect of multiple mutations in different parts of the molecule has rarely been explored, precluding a thorough investigation of conformational cross-talk.

One alternative to develop unimolecular polypharmacy agents is the use of selection methods, for example those based on phage-displayed peptide libraries (PDL)^24–27^. The defining feature of a PDL is the existence of a physical linkage between the peptide displayed on the surface (which determines the phenotype) and its encoding DNA (the genotype). Very large, diverse, random PDL can be constructed and selected, and the results of the screening can be rapidly decoded by DNA sequencing.

Peptide agonists and antagonists of cell membrane receptors have been successfully identified using this process^27–30^, including peptide agonists of G-protein-coupled receptors (GPCRs)^31–36^.

In 1997–98 Szardenings *et al*.^31^ and Rousch *et al*.^32^ first showed that phages bearing a GPCR agonist, the peptide hormones α-MSH or somatostatin, fused to the pIII or the pVIII protein could bind to cells expressing the respective receptor and most importantly, could activate it, acting as an agonist with lower^31^ or even comparable^32^ potency to the free peptide.

For the class B GPCRs, Yin *et al*. screened a PDL of Exendin-4 mutants (2–3 average mutations/molecule) on the N-terminal region including the ectodomain (aa 21–145) of the rat GLP-1 receptor (GLP1R)^34^. Sequencing of 60 clones from the third selection round (often called “biopanning”) led to the identification of a phage which bound the receptor fragment better than the wt peptide; the mutant phage featured the Glu16Val, Pro31Gln, and Ser33Asn mutations.

Koth *et al*. selected a PDL of glucagon mutants on the ectodomain of the GCGR, immobilized on a plate through a biotin tag^36^. In the library, residues 16–29 were “soft randomized” to be a mixture of 50% wt and 50% random amino acid. In the terminology first proposed by Schwyzer^37–39^, this segment corresponds to the “address” region of GCG, mostly responsible for receptor selectivity, while the N-terminal region represents the “message” segment, mostly responsible for receptor activation. Structural and modeling studies indicate that the “address” includes the key molecular determinants for binding to the receptor ectodomain, while the “message” is in contact with the 7TM membrane-embedded domain^36,40–42^.

Chen *et al*. selected a 12-amino acid random PDL on stable recombinant cells overexpressing the rat GLP1R^35^. Four rounds of selection enriched for GLP1R cell-binding phages. About 1% of the specifically bound phages (10 phages) were capable of activating the GLP1R, with the best peptide, KS-12, featuring high sequence homology to the N-terminal “message” region of GPL-1^35^. KS-12 showed EC_50_ of 0.8 μM, vs 0.8 nM of GLP-1 in the same assay.

Collectively, these studies established that (i) a peptide hormone – including GLP-1 – on the surface of a phage particle is capable of activating its receptor expressed on the cell surface; (ii) it is possible to identify active mutants both in the “message” and the “address” segments of the peptide ligands of class B GPCRs.

While all the above studies aimed at evolving a single receptor-specific peptide, there is no *a priori* reason why peptides able to activate more than one receptor should not be identified by a suitable selection strategy. We report here a proof-of-concept study of the selection of GCG/GLP-1 receptor co-agonists from a phage display library based on randomization of the glucagon sequence, sequentially selected on GCGR- and GLP1R-overexpressing cells. Two or three rounds of selection enabled the discovery of novel unimolecular co-agonists, with comparable *in vitro* potency to the reported rationally designed peptides^8,11^.

## Results

### Library design and cloning

**Figure 1:**
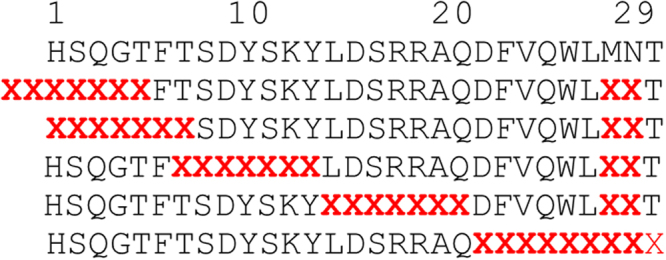
Design of the Phage Display Library (PDL). The Phage display library used to select GCGR/GLP1R co-agonists is composed of five sub-libraries, each one with nine randomized positions, indicated with a red bold X letter. Because of the chosen randomization scheme, each peptide displays 1–3 mutations distributed across the 9 randomized positions, with the remaining positions featuring the wt residue. For selection on GCGR^+^ and GLP1R^+^ cells, the five libraries were pulled (‘Library Mix’) and selected together.

To independently interrogate the “message” and “address” segments of GCG, the PDL was designed as the combination of four sub-libraries, each focusing on one of four seven-amino acid long consecutive segments (Fig. 1).

The two C-terminal residues 27 and 28, together with the N-terminal residues, exhibit more diversity among GCG and GLP-1 and are critical for the interaction of each hormone with the respective receptor^12^. These residues were mutagenized in all four sub-libraries.

Additionally, motivated by the results of Chen *et al*. showing that an additional N-terminal Lys residue was well tolerated for GLP1R agonism and offered increased resistance to DPP-IV^35,43^, we designed a fifth library exploring variants harboring a two amino acid extension at the GCG N-terminus.

Both GCG and GLP-1 are low picomolar ligands of the respective receptors, suggesting that their sequence is highly optimized; moreover, the work of Tschöp and co-coworkers had shown that relatively few mutations suffice to install dual- or even triple-agonism in the GCG sequence^8,9,11,12^. Therefore, in all the sub-libraries, instead of a standard full-randomization scheme (equal ratios of all amino acids except Cys) which would have predominantly provided variants with ≥4 mutations, we chose a randomization scheme where each mutagenized position retained a constant fraction of 45% wt residue: for a library with a targeted complexity 6 × 10^7^, this scheme produced variants with a smaller number (1–3) of mutations (Table 1), distributed through the 9 randomized positions, with the remaining positions featuring the wt residue.

**Table 1:** Distribution of the variants in a library of complexity 6 × 10^7^ with 45% wt amino acid at each of the 9 randomized positions.

No. of Mutations from GCG	Fraction	Cumulative Fraction	No. of physical clones	Theor. Diversity	Poisson estimate coverage	Number unique clones^1^	Average No. of duplicates
0 (wt)	0.08%	0.08%	4.54 × 10^4^	1.00	100.00%	1.00	45400.84
1	0.83%	0.91%	4.99 × 10^5^	1.62 × 10^2^	100.00%	1.62 × 10^2^	3082.77
2	4.07%	4.98%	2.44 × 10^6^	1.17 × 10^4^	100.00%	1.17 × 10^4^	209.32
3	11.60%	16.58%	6.96 × 10^6^	4.90 × 10^5^	100.00%	4.90 × 10^5^	14.21
4	21.28%	37.86%	1.28 × 10^7^	1.32 × 10^7^	61.91%	8.19 × 10^6^	1.56
5	26.00%	63.86%	1.56 × 10^7^	2.38 × 10^8^	6.34%	1.51 × 10^7^	1.03
6	21.19%	85.05%	1.27 × 10^7^	2.86 × 10^9^	0.44%	1.27 × 10^7^	≪1
7	11.10%	96.15%	6.66 × 10^6^	2.20 × 10^10^	0.03%	6.66 × 10^6^	≪1
8	3.39%	99.54%	2.03 × 10^6^	9.92 × 10^10^	0.00%	2.03 × 10^6^	≪1
9	0.46%	100.00%	2.76 × 10^5^	1.98 × 10^11^	0.00%	2.76 × 10^5^	≪1
Sum:	100%		6.00 × 10^7^			4.55 × 10^7^	

^1^Based on Poisson estimate for coverage of theoretical diversity (fractional completeness).

After cloning each of the five sub-libraries into the recipient vector and generation of the phage progeny, a small screening using 96-well plates indicated that for all the five libraries the designed amino acid composition was correctly represented.

### Selection of the PDL on GCGR^+^ and GLP1R^+^ cells

**Figure 2:**
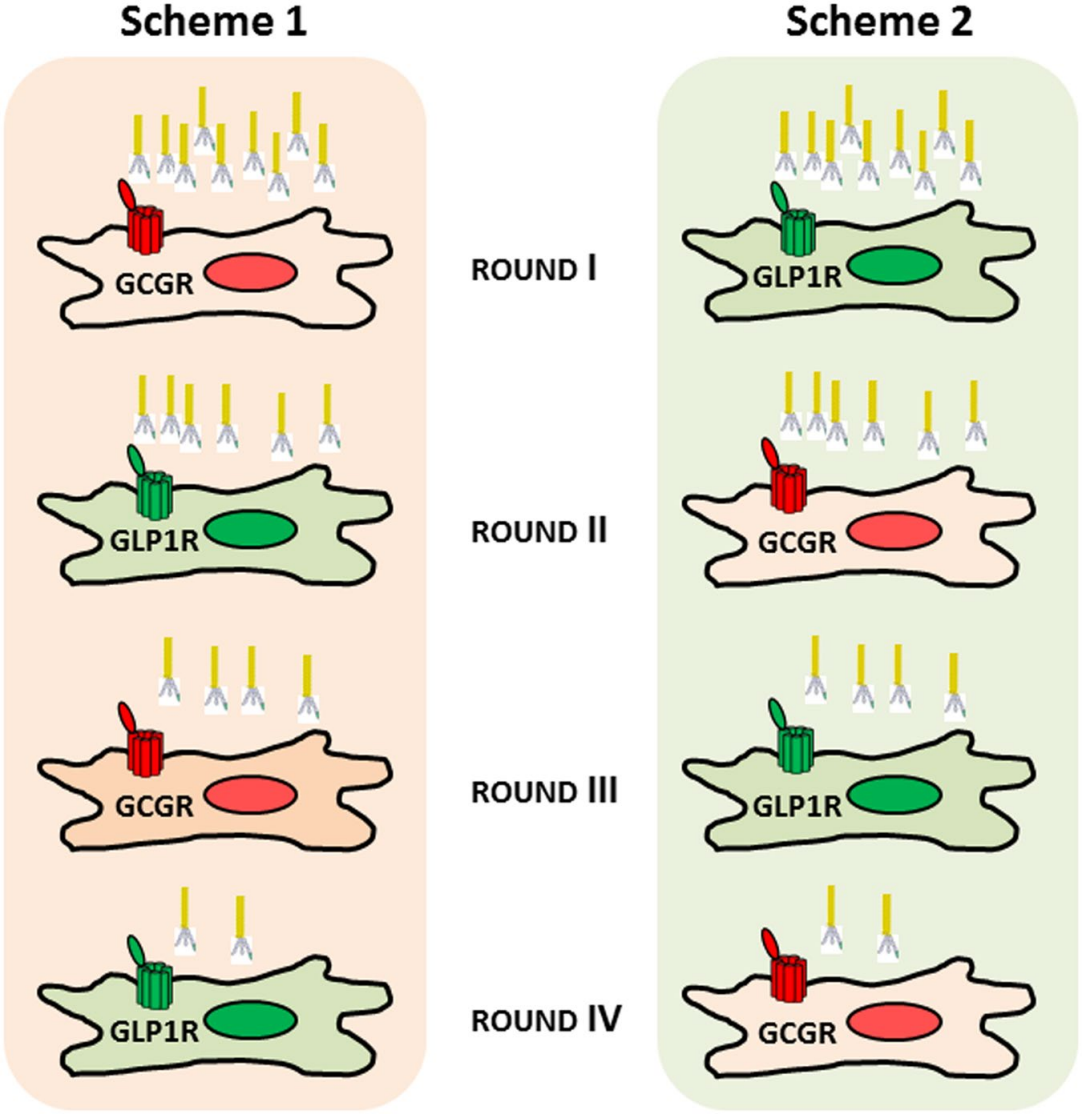
Selection Schemes for the PDL. The Library Mix was selected alternatively on GCGR^+^ and GLP1R^+^ HEK293 overexpressing cells.

**Figure 3:**
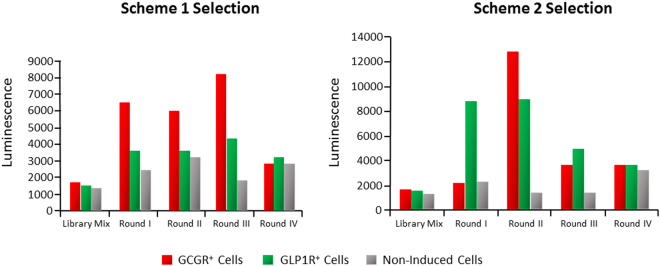
Pooled selected phages activate both the GCGR and the GLP1R. Activity of phage pools from the four rounds of selection according to Schemes 1 and 2 in the c-AMP activity assay on GCGR^+^, GLP1R^+^, and parental cells.

To identify phages able to activate both the GCGR and the GLP1R, a Library Mix was prepared by combining into a single pool an equivalent amount of phages from each sub-library. Two selection schemes were used, alternating four rounds of selection on GCGR- and GLP1R-overexpressing cell lines (Fig. 2). The pools of phages coming from the four rounds of selection were tested, together with the unselected libraries, by cAMP-activity assay on naïve, GCGR^+^ and GLP1R^+^ overexpressing cells. As shown in Fig. 3, a common feature of the two selection schemes was an immediate increase of activity on the first receptor screened (GCGR for Scheme 1 and GLP1R for Scheme 2). At the second round the two schemes diverged, with co-agonism appearing more readily in Scheme 2, possibly indicating that it is easier to install GCGR agonism into a GLP-1 backbone than *vice versa*. Another common feature of the two schemes was that co-agonism was quickly optimized after 2–3 rounds, and further rounds lead to decline of reactivity on both receptors. This is probably due to the fact that the selection process is driven by binding but our screening is based on activity, and the two features do not necessarily overlap.

### Individual Phage Clones activate the GCGR and GLP1R

The purpose of the selection was to identify peptides able to *activate* both the GCGR and the GLP1R. Selection from the PDL, however, depended on the ability to *bind* the two receptors. We randomly picked 192 clones from Round III of selection Scheme 1 (Selection 1-III) and 192 clones from Round II of selection Scheme 2 (Selection 2-II), amplified them in 96-well plates, and tested for activation of GCGR^+^ and GLP1R^+^ cells. Since high-throughput amplification does not allow control over the phage concentration in the supernatant, this analysis aimed only at a qualitative confirmation of the collective properties of the selected pools: we defined positivity as signal above an arbitrarily defined threshold, and found that 64/192 (33%) and 80/192 (42%) clones were agonists of both GCGR and GLP1R for Selection 1-III and Selection 2-II, respectively (Figure S1). 104 positive clones were individually grown at larger scale, to obtain phage supernatants with a titer ≥5 × 10^10^ TU/mL, and these were again tested on GCGR^+^ and GLP1R^+^ cells. 12 phages from Selection 1-III and 17 phages from Selection 2-II were found to potently activate both receptors, while 2 and 4 phages, respectively, were potent agonists of the GCGR only. Overall, 29/384 (7.5%) of randomly picked clones were co-agonists, and a further 6/384 (1.5%) were agonists of a single receptor. The high percentage of binding-activating phages validates our strategy of using a conservative design for the library, maintaining in all phage clones both the ‘address’ and ‘message’ regions of the hormone, and limiting the allowed number of mutations per molecule. For comparison, in the selection by Chen *et al*. of a totally random 12-aa PDL on rat GLP1R, only about 1% of the GLP1R cell-binding phages were agonists of the receptor^35^.

### Phage-derived peptides are potent, balanced co-agonists of the GCGR and GLP1R

The sequence of the agonist and co-agonist phages was determined, and 35 corresponding peptides were synthesized. The peptides were tested on GCGR^+^ and GLP1R^+^ cells, and the results are reported in Table 2. Only two peptides were inactive/poorly active (i.e. showed EC_50_ > 20 nM) on both receptors. Among the others, 8 peptides were found to be extremely potent co-agonists, with EC_50_ ≤ 30 pM on each receptor, and a further 10 peptides showed EC_50_ ≤ 80 pM on the two receptors, favorably comparing with the best rationally designed glucagon/GLP-1 co-agonists reported in the literature^8,11^. Importantly, 11/18 co-agonists displayed a GLP1R/GCGR EC_50_ ratio between 0.5 and 1.5, which according to the study of Day *et al*.^11^, represents the optimal balance of co-agonism to maintain full glycemic control while maximizing weight loss.

**Table 2:** Activity of phage-derived peptides on GCGR and GLP1R.

Peptide	Sequence^1^	EC_50_ GCGR (nM)	EC_50_ GLP1R (nM)	GCGR/GLP1R EC_50_ ratio
1	HS**V**G**N**F**W**SDYSKYLDSRRAQDFVQWLMLT	8.445 ± 2.667	16.68 ± 10.30	0.51
2	HSQGTFTSDYSKY**VED**RRA**H**DFVQWLMNT	2.239 ± 0.334	0.184 ± 0.006	12.17
3	HSQGTFTSDY**R**KYLD**E**R**A**A**W**DFVQWLMNT	1.351 ± 0.173	0.427 ± 0.022	3.16
4	HSQGTFTSDYSKYLD**IG**RAQDFVQWL**L**NT	0.026 ± 0.002	1.328 ± 0.209	0.02
5	HSQGTFTSDYSKYLDS**LM**AQDFVQWLM**S**T	0.025 ± 0.001	0.133 ± 0.032	0.19
6	HSQGTFTSDYSKYLD**W**RRAQDFVQWL**L**NT	0.032 ± 0.013	0.063 ± 0.034	0.51
7	HSQGTFTSDY**I**K**L**LDSRRAQDFVQWLMNT	0.301 ± 0.216	78.32 ± 93.80	0.01
8	HSQGTFTSDYSKYLD**A**RRAQDFVQWL**IR**T	0.038 ± 0.010	0.017 ± 0.001	2.23
9	HSQGTFTSDYSKYLD**V**RRAQDFVQWLMNT	0.022 ± 0.007	0.064 ± 0.015	0.34
10	HSQGTFTSDYSKYLD**EL**RA**Y**DFVQWLMNT	0.057 ± 0.021	0.038 ± 0.009	1.50
11	HSQGTFTSDYSKYLDSRRA**H**DFVQWL**L**NT	0.018 ± 0.005	0.018 ± 0.001	1
12	HSQGTFTSDYSKYLDSRRAQDFVQWLMN**P**	0.040 ± 0.019	0.124 ± 0.027	0.32
13	HSQGTFTSDYSKYLDSRRAQDFVQWL**I**N**Y**	0.078 ± 0.050	0.039 ± 0.008	2
14	**IN**H**E**Q**WA**FTSDYSKYLDSRRAQDFVQWLMNT	2.604 ± 3.682	16.68 ± 10.30	0.16
15	**A**S**MF**TF**F**SDYSKYLDSRRAQDFVQWLM**L**T	>20	>200	nd
16	HSQGTF**L**SDYSK**L**LDSRRAQDFVQWLM**Q**T	0.131 ± 0.060	11.22 ± 10.56	0.01
17	HSQGTF**LH**DY**YY**YLDSRRAQDFVQWLM**D**T	0.527 ± 0.315	>200	nd
18	HSQGTFTSDYSKYLDS**I**RAQDFVQWLM**D**T	0.037 ± 0.011	0.056 ± 0.012	0.66
19	HSQGTFTSDYSKYLDSRRAQDFV**D**WLMN**E**	0.014 ± 0.001	0.055 ± 0.006	0.25
20	HSQGTFTSDYSKYLDSRRAQDFVQWL**I**NT	0.031 ± 0.004	0.017 ± 0.002	1.82
21	**KALG**QFTFTSDYSKYLDSRRAQDFVQWLMNT	4.184 ± 1.134	14.83 ± 11.42	0.28
22	HSQGTF**F**SDYS**HW**LDSRRAQDFVQWLMNT	0.029 ± 0.001	>200	nd
23	HSQGTFTSDYSKYLD**W**RRAQDFVQWL**Q**NT	0.019 ± 0.001	0.017 ± 0.001	1.12
24	HSQGTFTSDYSKYLDS**K**RA**H**DFVQWL**L**NT	0.026 ± 0.001	0.018 ± 0.001	1.44
25	HSQGTFTSDYSKYLDSRRAQDF**WID**LMNT	>20	>20	nd
26	HSQGTFTSDYSKYLDSRRAQDFV**MTS**MNT	>200	8.957 ± 1.840	nd
27	HSQGTFTSDYSKYLDSRRAQDFV**E**WLMN**N**	0.015 ± 0.003	0.029 ± 0.001	0.52
28	HSQGTFTSDYSKYLDSRRAQDFV**D**WL**I**N**S**	0.016 ± 0.001	0.007 ± 0.001	2.28
29	HS**H**GTFTSDYSKYLDSRRAQDFVQWLM**T**T	0.032 ± 0.003	0.080 ± 0.006	0.40
30	HSQG**I**F**F**SDYSKYLDSRRAQDFVQWLMNT	0.026 ± 0.004	>200	nd
31	HSQGTFTSDYS**W**YLDSRRAQDFVQWLMNT	0.032 ± 0.011	0.044 ± 0.004	0.73
32	HSQGTFTSDYSKYLD**MQ**RA**H**DFVQWLMNT	0.014 ± 0.001	0.022 ± 0.001	0.64
33	HSQGTFTSDYSKYLDSR**M**A**Y**DFVQWLMNT	0.079 ± 0.033	0.059 ± 0.003	1.34
34	HSQGTF**F**SDYSKYLDSRRAQDFVQWL**LE**T	0.027 ± 0.005	51.94 ± 57.71	0.001
35	HSQGTFTSDYSKYLDSRRAQDFVQWL**LDS**	0.0019 ± 0.005	0.018 ± 0.001	0.10

^1^All peptides C-terminal carboxyamide; bold, mutations from the native GCG sequence.

Inspection of the sequence of the peptides of Table 2 shows that the mutations leading to balanced co-agonism are distributed throughout the glucagon backbone, sometimes with cross-talk between the N-terminal and the C-terminal region (Peptide #34, Thr7 → Phe + Met27Asn28 → Leu27Glu28). This confirms that selection from the library has enabled exploration of a larger chemical space than the one sampled by rational design and accordingly, the mutations found in the phage-derived sequences show very little, if any, overlap with the amino acid changes featured in the sequence of the co-agonists reported in the literature^8,11^.

We may conclude that limited sampling of the sequences of the selected phages was sufficient to identify numerous co-agonists with the desired *in vitro* properties.

### From Phage-derived Peptides to Peptide Therapeutics

**Figure 4:**
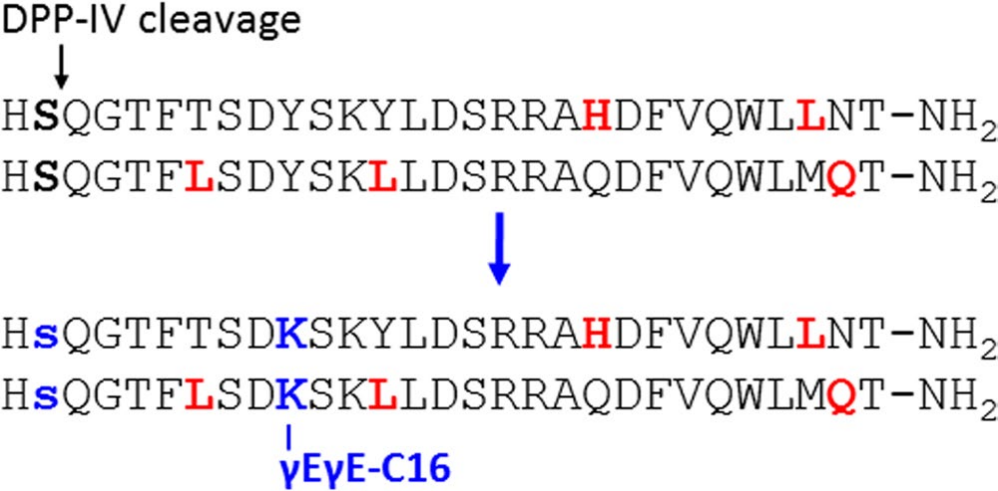
From Phage-derived Peptide to Peptide Therapeutic. Illustrated are the changes introduced into peptides #11, and #16, Table 2, to impart drug-like properties: Blue, changes introduced, s = D-Ser Red, mutations from the native GCG sequence derived from selection of the PDL. For a detailed explanation of the chosen changes, see text.

One potential liability of using a biological tool to identify peptide leads is the need to install drug-like properties onto the phage-derived sequences, i.e. to protect the peptide sequence from enzymatic degradation and rapid renal excretion, which typically conjure to drastically reduce peptide half-life *in vivo*. However, the rapidly accumulating experience on successful peptide lead-to-therapeutic transition^44,45^ suggests that this liability may not be a major one. To prove that this is the case, we analyzed the sequence of one of the phage-derived peptides with the best co-agonist *in vitro* profile, peptide #11, and of a strongly glucagon-biased agonist, peptide #16, since interest in improved glucagon agonists is raising^46–48^. We corrected the key liability common to both peptides, the presence of His1-Ser2 dipeptidyl peptidase IV (DPP-IV) cleavage site^49^, with the Ser2 → (D)-Ser2 substitution^23,50^, and installed a PK-modifier moiety – γGlu-γGlu-C16 – used in GLP1R agonists like liraglutide^51^ onto the side chain of a Lys residue substituted for Tyr in position 10 (Fig. 4).

These changes had indeed the expected outcome, since the resulting engineered peptides, #36–37, maintained comparable potency on both GCGR and GLP1R to the parent phage-derived peptides (Table 3).

**Table 3:** Comparison of engineered and parent phage-derived peptides.

Peptide	Sequence	EC_50_ hGCGR (nM)	EC_50_ hGLP1 (nM)
11	HSQGTFTSDY SKYLDSRRA**H**DFVQWL**L**NT	0.018 ± 0.005	0.018 ± 0.001
36	H**s**QGTFTSD**K**(**γEγEC16**) SKYLDSRRA**H**DFVQWL**L**NT	0.023 ± 0.004	0.050 ± 0.006
16	HSQGTF**L**SDY SK**L**LDSRAAQDFVQWLM**Q**T	0.131 ± 0.060	11.22 ± 10.56
37	H**s**QGTF**L**SDK(**γEγEC16**) SK**L**LDSRAAQDFVQWLM**Q**T	0.037 ± 0.004	23.48 ± 7.599

^1^All peptides C-terminal carboxyamide; s = D-Ser; γE = γ-glutamic acid, C16 = palmitic acid; bold, changes from the native GCG sequence.

## Discussion

In this manuscript, we describe a strategy for the identification of dual agonists of the GCGR and GLP1R through sequential selection of a PDL on cells expressing either receptor. This strategy led to the discovery of several novel dual agonists, unrelated to those previously described and with comparable potency. Moreover, we could show that these peptides could be made more “drug-like” by predictable changes, like eliminating enzymatic cleavage sites and introducing known PK-modifying moieties, an important step in the transition from phage-derived sequence to preclinical candidate.

A number of considerations apply to the output of the selection.

First, the selection was necessarily based on binding, not activation of the receptors. We used a number of features to reduce the number of selected phages which bind to, but do not activate the receptors, namely: (i) We reduced the diversity to 1–3 mutations per sequence, (ii) We reduced the number of selection cycles, and (iii) We alternated the order of the receptor-positive cells in the selection. Moreover, in three out of five libraries the N-terminal “message”, mainly responsible for receptor activation, was not randomized. Nevertheless, when we tested the peptides from the selected phages (Table 2), some did not activate one (#17, #26, #30) or both (#15, #25) receptors.

Second, when comparing the activity of the individual phage clones with the activity of the corresponding free peptides, one should take into account that although both the phage-displayed peptide and the free peptide are able to engage the receptor, their ability to induce downstream signaling^48–50^ may differ. Moreover, the situation may vary for the two receptors, resulting in a different relative potency (receptor “balance”) for the phage-displayed vs. the free co-agonist. Therefore, the selection process represents an approximation of the natural situation, and may be only indicative of the *in vivo* pharmacology of the individual phage-derived peptides.

Third, the fact that the selection provides a panel of peptides with variable balance represents a major advantage of the strategy, since it enables establishing an *in vitro*/*in vivo* pharmacology correlation, as elegantly shown by Day *et al*.^11^: studies in diet-induced obese (DIO) mice chronically treated with GCGR/GLP1R co-agonists with a different balance showed that maximal weight loss devoid of any sign of hyperglycemia was achieved with comparable *in vitro* potency at the two murine receptors, while further increase of potency at the GCGR increased weight loss but led to elevation of blood glucose^11^. The panel of peptides in Table 2 shows examples of GCGR-biased (peptides #5, #7, #34), GLP1R-biased (peptides # 2, #3), and balanced (all the other) co-agonists, which could be used for the experiments performed by Day *et al*., after engineering for *in vivo* studies as shown for peptides # 36–37 (Fig. 4).

Finally, this strategy appears of general value for the discovery of optimized polypharmacology paradigms across several metabolically-related hormones. For example, a selection cascade including GIPR+ cells in addition to GCGR+ and GLP1R+ cells might enable the identification of a triple-agonist as reported by Finan *et al*.^12^.

## Methods

### Library design and generation

Five sub-libraries were designed covering the whole wt GCG sequence, each with nine positions mutated and with a targeted complexity of 6 × 10^7^ clones (Fig. 1). To ensure that all possible variants with one, two or three mutations were present in the sub-libraries, we used the “doping-style” design depicted in Table 1, and the randomized positions in the oligonucleotides were introduced using codon-focused trimer-blocks^26,52^. At each mutated position, 45% of the variants maintained a codon for the wt sequence and the remaining 55% of the variants represented an equal mixture of codons for the 18 non-wt residues (always excluding Cys). The presence of NcoI and NotI restriction sites necessary for cloning of the sub-libraries into the pCANTAB6 phagemid vector was minimized to less than 0.13% by choosing appropriate codon trimer-blocks. Synthesis of oligonucleotides was performed by Ella Biotech GmbH (Munich, Germany) utilizing the desired mixture of pre-build codon-focused trimer-blocks at each randomized position. Double-strand DNA for cloning each sub-library into the pCANTAB6 vector was generated by primer extension, combining the relative forward and reverse oligo for each library (Figure S2). 600 pmol of forward and reverse oligos were denatured at 95 °C for 3 min, annealed at 68 °C for 30 sec and extended for 30 sec at 72 °C in the presence of 0.2 mM dNTS,1 unit of Phusion High –Fidelity DNA Polymerase (#F-530, Thermo Scientific) and 1.5 M Betaine solution (# B0300, Sigma Aldrich).

Once checked for size on agarose gel, the fragments were digested with NcoI-NotI (NcoI #R0193S and NotI #R0189L Bio Labs), purified through Qiaquick nucleotide Removal Kit (Qiagen), quantified by OD_260nM_ and ligated into NcoI-NotI pCANTAB6 phagemid. Preparative ligations were assembled by mixing vector and inserts at 1:5 molar ratio and incubated o/n at 16 °C with T4 DNA ligase (M02024, Bio Labs). The ligation product was desalted and transformed into electrocompetent *E*.*coli* XL1Blue cells (Agilent Technologies) using an electroporator (Bio-Rad, Hercules, CA) at 1.8 kV, 25 μF and 200 Ω. Transformed bacteria were plated on 2XTY/Amp (100 µg/mL)/2% Glucose 23 × 23 square plates and incubated o/n at 37 °C. The day after the plates were scraped to harvest bacteria in 10 ml 2XTY Amp (100 µg/ml)/2% Glucose containing 17% glycerol, and stored at −80 °C. For each library, 6 × 10^7^ individual clones were collected, for a cumulative complexity of 3 × 10^8^ for the five sub-libraries. Phage libraries were prepared by super-infection of transformed bacteria with 10 MOI of M13K07 helper phage, purified through CsCl gradient, according to standard protocols, and titrated as plaque-forming units (pfu) and transforming units (tu)/mL. The Library Mix was prepared by mixing an equivalent amount of phages from each sub-library.

To obtain positive control phages for the screening, DNA sequences coding for the sequence of Glucagon and GLP-1 were subcloned as a NcoI-NotI fragment into pCANTAB6 phagemid, fused with the N-Terminus sequence of M13 bacteriophage coat protein pIII. Once obtained, the GCG and GLP-1 phagemids were converted to phage particles, purified on a CsCl gradient and titrated.

### Selection of GCG/GLP1 co-agonists from phage libraries

10^11^ phages of the Library Mix were used for the first round of selection on GCGR^+^ and GLP1R^+^ overexpressing HEK293 cells, after depletion on naïve cells. Four rounds of selection were carried out, with two different selection schemes: in Scheme 1, the first round was made on GCGR^+^ cells, followed by a second round on GLP1R^+^ cells, a third round on GCGR^+^ cells, and a fourth round on GLP1R^+^ cells; in Scheme 2, the order of the cells was inverted (Fig. 2). Briefly, phage particles were blocked in 0.5 mL of phosphate-buffered saline (PBS) containing 3% non-fat dry milk (MPBS) for 30 min at RT, then incubated with 10^7^ naïve cells previously resupended in MPBS for 1 additional hour in a rotary mixer. Cells were pelleted, the depleted phage supernatant was recovered, then incubated with either GLP1R^+^ or GCGR^+^ cells, resupended into 0.5 mL of MPBS for 1hr in a rotary mixer. After incubation, the mixture of cells and phages was centrifuged for 1 min at 2000 rpm, the supernatant was discarded, and the pellet was washed 6 times with PBS. For the elution step, the pellet was resuspended in 100 mM HCl (500 µL) for 15 min at RT in a rotary mixer, then neutralized with 100 mM TrisHCl pH 7.5. Eluted phages were transferred in 50 mL Falcon tube, 10 mL of TG1 cells were added and the mixture was incubated for 1hr at 37 °C at 150 rpm. TG1 infected cells were plated out onto 2XTY Amp/Glu 23 × 23 square plate and incubated overnight (o/n) at 30 °C. The day after the selected pool of phages was rescued by scraping bacteria from bioassay plate in 10 mL of 2XTY Amp/Glu. 50 µL of the scraping was inoculated in 50 mL 2XTY Amp/Glu and grown at 37 °C to O.D._600nm_ = 0.5 in a shaker at 180 rpm. M13K07 helper phage was added to bacteria at 10 multiplicity of infection (MOI), and incubated at 37 °C for 30 min in static and 30 min in agitation (120 rpm). The culture was centrifuged for 10 min at 3200 rpm, the supernatant was removed, the pellet was resuspended in 50 mL 2XTY Amp (100 µg/mL)/Kan (25 µg/mL) and grown o/n at 25 °C at 210 rpm. The day after the culture was centrifuged at 4200 rpm for 45 min at 4 °C, the supernatant was recovered and used to perform the next round of selection.

### Screening by cAMP activity assay

The pools of phage supernatants deriving from each round of selection were first tested for activity to determine which round of selection to screen further as single clone in the 96 well format. Phages from each round of selection were screened for activity both as a pool and as single clone phage supernatants. From each round of selection, about 200 single clones were isolated and prepared as phage supernatants in 96 well format. Once prepared as above described, phages were precipitated from the supernatants by adding 1/3 volume of 20% PEG8000/2.5 M NaCl o/n at 4 °C, centrifuged for 45 min at 4200 rpm at 4 °C, and resuspended in 1/10 volume of PBS. Both the phage pools and the single phages were tested with a cAMP activity assay (HitHunter cAMP XS+ Assay *DiscoverX*, cat#90-0075), according to the manufacturer’s instructions. Naïve, GCGR^+^ and GLP1R^+^ overexpressing cells, were harvested, counted and re-suspended in Assay Buffer (AB, 7.5% BSA, 10% FBS, PBS + R01724 phosphatase inhibitor). 8 µL of each phage supernatant was diluted 1:5 into AB and incubated for 3 hr at 25 °C with 30.000 cells/well. The specific activity was determined as luminescence with a Safire2 TECAN instrument. Phages able to activate both the GCGR and GLP1R were isolated, the phagemids were extracted and subjected to DNA sequencing. Peptides corresponding to the sequence of the selected phages were synthesized for further testing.

### Peptide Synthesis and Testing for activity

Peptides were synthesized by solid phase synthesis using Fmoc/*t*-Bu chemistry on a Symphony peptide synthesizer (Protein Technologies Inc.) on a Rink-amide PEG-PS resin (Champion, Biosearch Technologies, 0.28 mmol/g), as previously described^10,23^. The peptides were purified by reverse-phase HPLC using a Waters X-Bridge C18 column (50 × 150 mm, 5 µm, 130 Ǻ) and 0.1% TFA in water and 0.1% TFA in acetonitrile as eluents. Analytical HPLC was performed on an Acquity UPLC Chromatograph (Waters) with a BEH130 C18 or a BEH300 C4 Acquity column (2.1 × 100 mm, 1.7 µm, Waters) at 45 °C, using water, 0.1% TFA and acetonitrile, 0.1% TFA as solvents. The peptides were characterized by electrospray mass spectrometry on an Acquity SQ Detector. Analytical data for peptides are given in the Supporting Information.

Peptides were tested *in vitro* for activity using cAMP assays on both the GCG and GLP-1 receptors as previously described^10^. Briefly, a 2 μM solution of peptide was prepared, and 4-fold serial dilutions were made. 150 nL of the above titration was then dispensed into an Optiplate384 using an Echo555. CHO-JumpIn cells, stably transfected with human GLP-1 receptor (GLP1R) or glucagon receptor (GCGR), were counted with a cell counter (Countess;Invitrogen) and resuspended in assay buffer containing PBS(Gibco # A12858), 0.1% BSA, 100 μM RO 20-1724 and 20% species specific serum. The HitHunter® cAMP Assay kit calibration curve was prepared following the manufacturer’s protocol (DiscoveRx-90-0075L). 30,000 cells/well were added to the peptides, spun briefly at 500 rpm for 15 s and incubated at RT in the dark for 1 hr. The assay was developed as per the manufacturer’s instructions. cAMP accumulation due to GLP1R agonism or GCGR agonism was measured using an EnVision platereader (PerkinElmer), and calibrated using the cAMP standard curve. Data were analyzed using Assay Data Analyzer (ADA).

### Data Availability

All data generated or analyzed during this study are included in this published article and its Supplementary Information file.

## Electronic supplementary material

Supplementary Information
