# Supplementary material for: Polypharmacy through Phage Display: Selection of Glucagon and GLP-1 Receptor Co-agonists from a Phage-Displayed Peptide Library
Source: Sci Rep. 2018 Jan 12;8:585. doi: 10.1038/s41598-017-18494-5 (PMC5766609; doi:10.1038/s41598-017-18494-5)
Supplement: Supplementary file 1 — Supplementary Information [file 41598_2017_18494_MOESM1_ESM.pdf]

Supplementary Information for

Polypharmacy through Phage Display: Selection of Glucagon and GLP-1 Receptor Co-agonists from a Phage-Displayed Peptide Library

Anna Demartis, Armin Lahm, Licia Tomei, Elisa Beghetto, Valentina Di Biasio, Federica Orvieto, Francesco Frattolillo, Paul E. Carrington, Sheena Mumick, Brian Hawes, Elisabetta Bianchi, Anandan Palani, and Antonello Pessi

Table S1
Figure S1
Figure S2
Analytical data for all the peptides of Tables 2 and 3.

Table S1. Distribution of the variants in a library of complexity 6×10^7 with 45% wt amino acid at each of the 9 randomized positions.

No. of Mutations	Fraction	Cumulative Fraction	No. of physical clones	Theor. Diversity	Poisson estimate coverage	Number unique clones[2]	No. of duplicates[3]
0 (wt)	0.08%	0.08%	4.54×10^4	1.00	100.00%	1.00	45400.84
1	0.83%	0.91%	4.99×10^5	1.62×10^2	100.00%	1.62×10^2	3082.77
2	4.07%	4.98%	2.44×10^6	1.17×10^4	100.00%	1.17×10^4	209.32
3	11.60%	16.58%	6.96×10^6	4.90×10^5	100.00%	4.90×10^5	14.21
4	21.28%	37.86%	1.28×10^7	1.32×10^7	61.91%	8.19×10^6	1.56
5	26.00%	63.86%	1.56×10^7	2.38×10^8	6.34%	1.51×10^7	1.03
6	21.19%	85.05%	1.27×10^7	2.86×10^9	0.44%	1.27×10^7	<<1
7	11.10%	96.15%	6.66×10^6	2.20×10^{10}	0.03%	6.66×10^6	<<1
8	3.39%	99.54%	2.03×10^6	9.92×10^{10}	0.00%	2.03×10^6	<<1
9	0.46%	100.00%	2.76×10^5	1.98×10^{11}	0.00%	2.76×10^5	<<1
Sum:	100%		6.00×10^7			4.55×10^7	

[1]From GCG sequence; [2]Based on Poisson estimate for coverage of theoretical diversity (fractional completeness); [3]Average

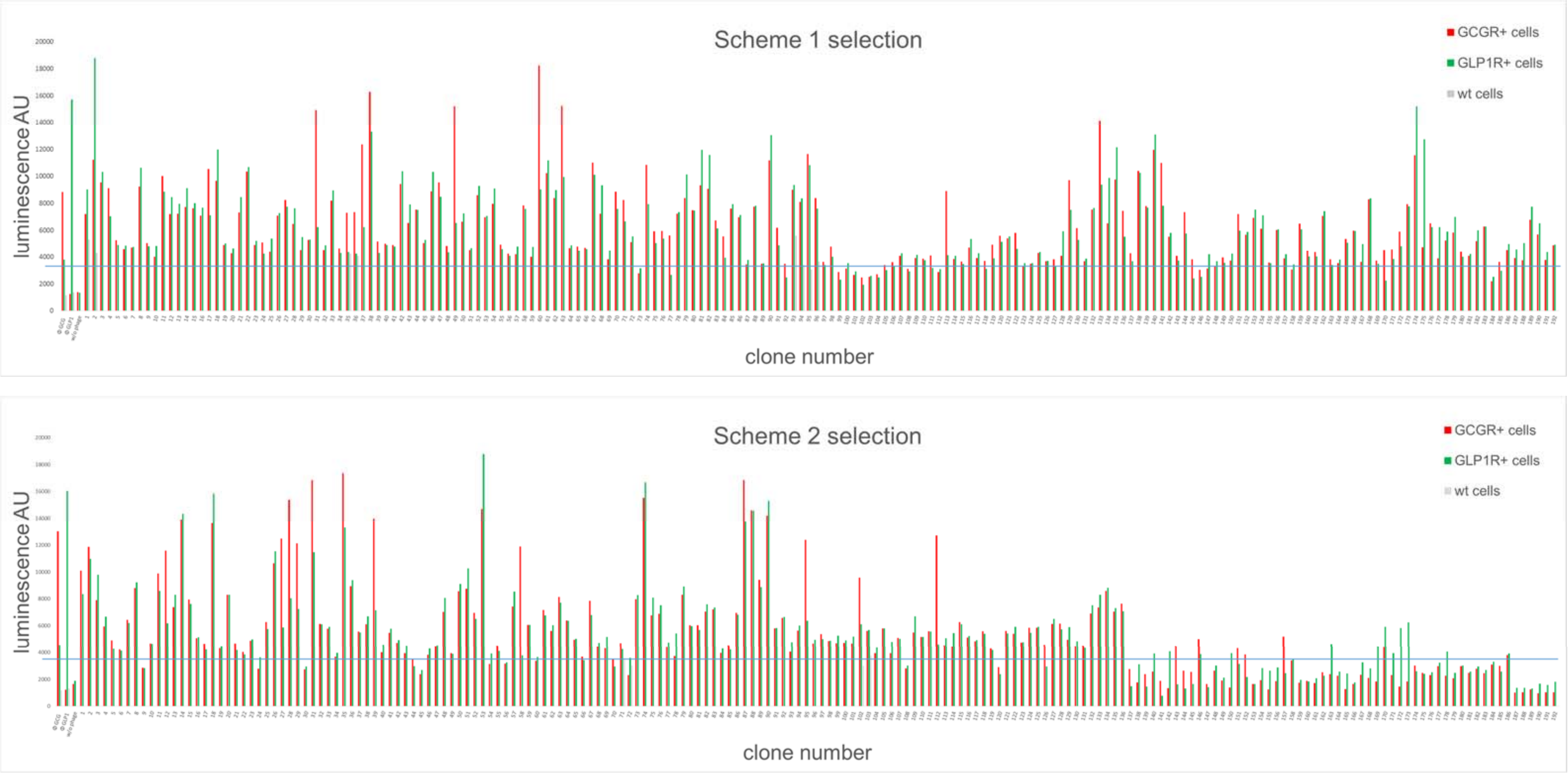

Figure S1. **Single phages activate both the GCGR and the GLP1R.** Screening by cAMP activity assay on GCGR+ and GLP1R+ cells of 96 single phage clones from Round-III of Scheme1 selection, and 96 single phage clones from Round-II of Scheme 2 selection

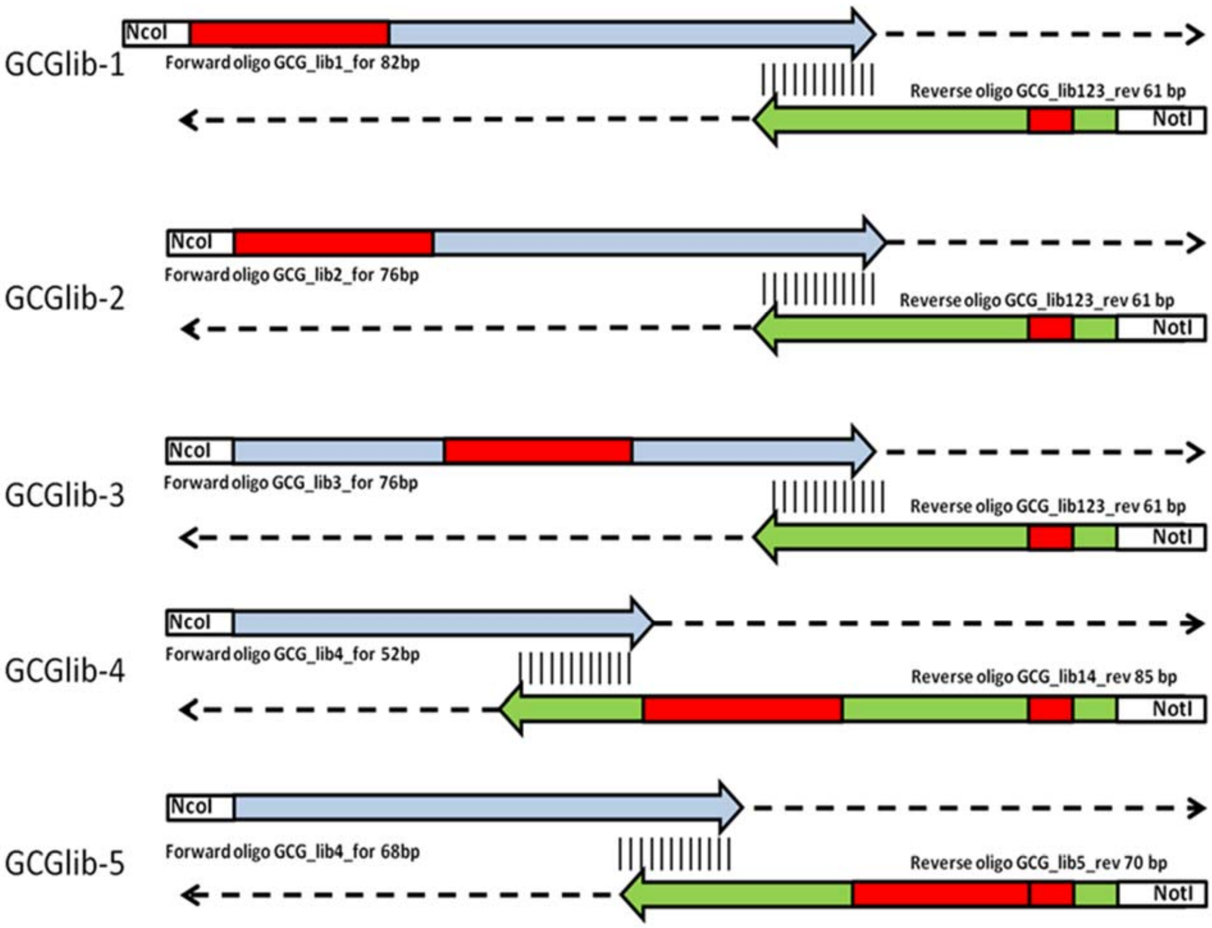

Figure S2. Construction of the Genes for the PDL. The scheme describes the oligonucleotides used to assemble the five sub-libraries. At each mutagenized position, the respective oligonucleotide contains 45% wt codon and an equal mixture of 18 non-wt codons, excluding Cys

Table2: Peptide 1: upper panel: UPLC-UV Analysis on Waters Acquity BEH C18 column (2.1x 100 mm, 1.7 μm, 130 A), Waters TUV detector λ= 214 nm, solvent A= H2O, 0.1% TFA; B: CH3CN, 0.1% TFA; gradient of B 20%-20% (1')-60% (5')-80% (1'). Lower panel: ESI mass spectrometry on Waters SQ detector: MW theoretical =3549.98; MW calculated =3549.40

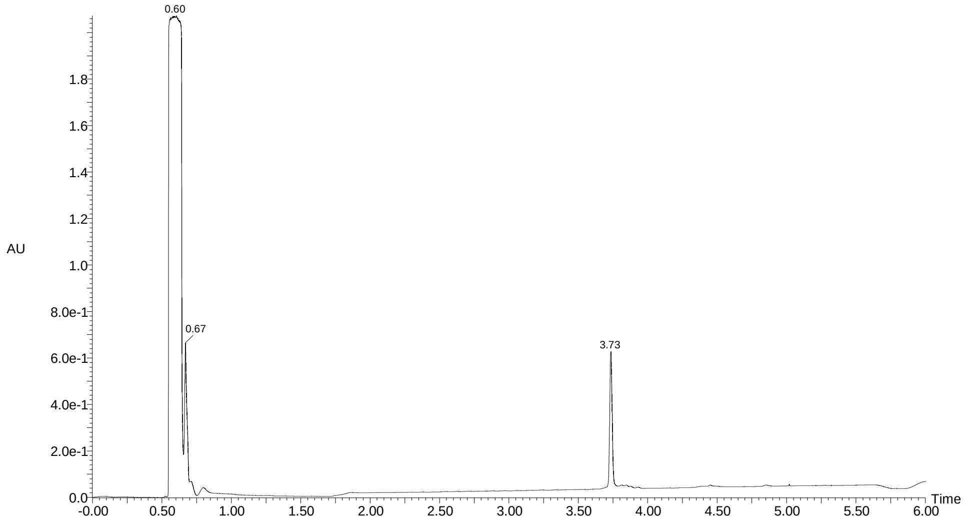

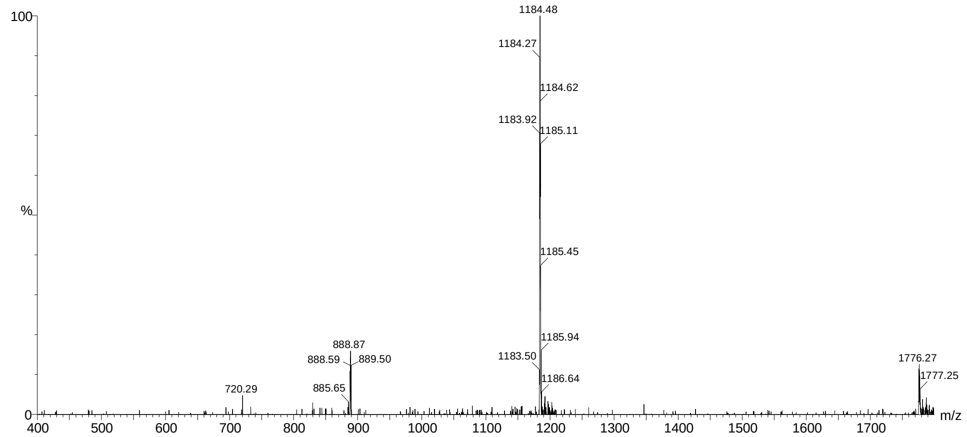

Table2: Peptide 2: upper panel: UPLC-UV Analysis on Waters Acquity BEH C18 column (2.1x 100 mm, 1.7 μm, 130 A), Waters TUV detector λ= 214 nm, solvent A= H2O, 0.1% TFA; B: CH3CN, 0.1% TFA; gradient of B 20%-20% (1')-60% (4'). Lower panel: ESI mass spectrometry on Waters SQ detector: MW theoretical= 3518.83; MW calculated = 3519.4

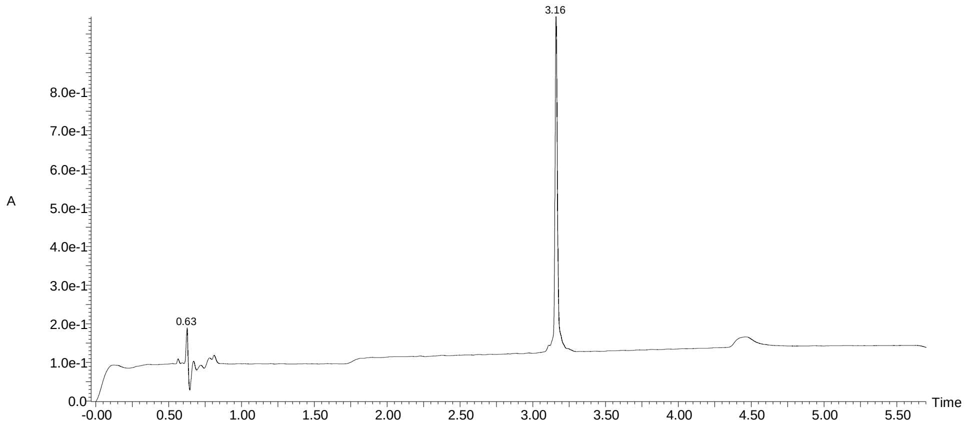

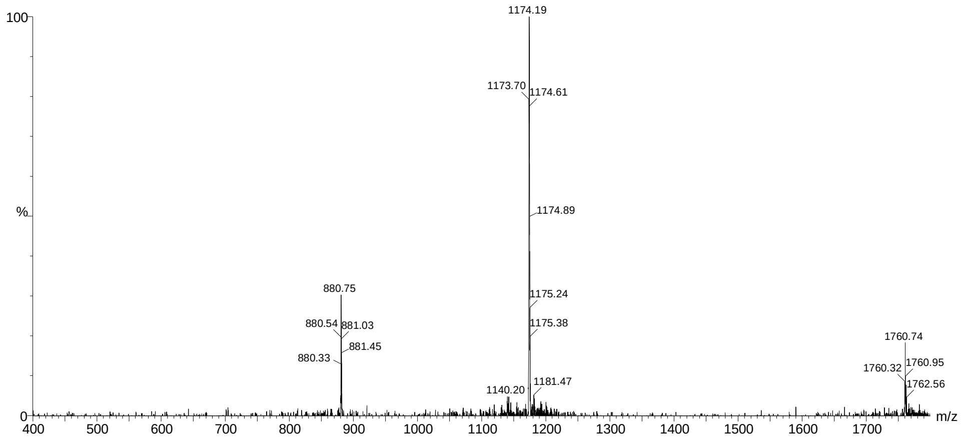

Table2: Peptide 3: upper panel: UPLC-UV Analysis on Waters Acquity BEH C18 column (2.1x 100 mm, 1.7 μm, 130 A), Waters TUV detector λ= 214 nm, solvent A= H2O, 0.1% TFA; B: CH3CN, 0.1% TFA; gradient of B 20%-20% (1')-60% (4'). Lower panel: ESI mass spectrometry on Waters SQ detector: MW theoretical= 3565.93; MW calculated = 3566.58

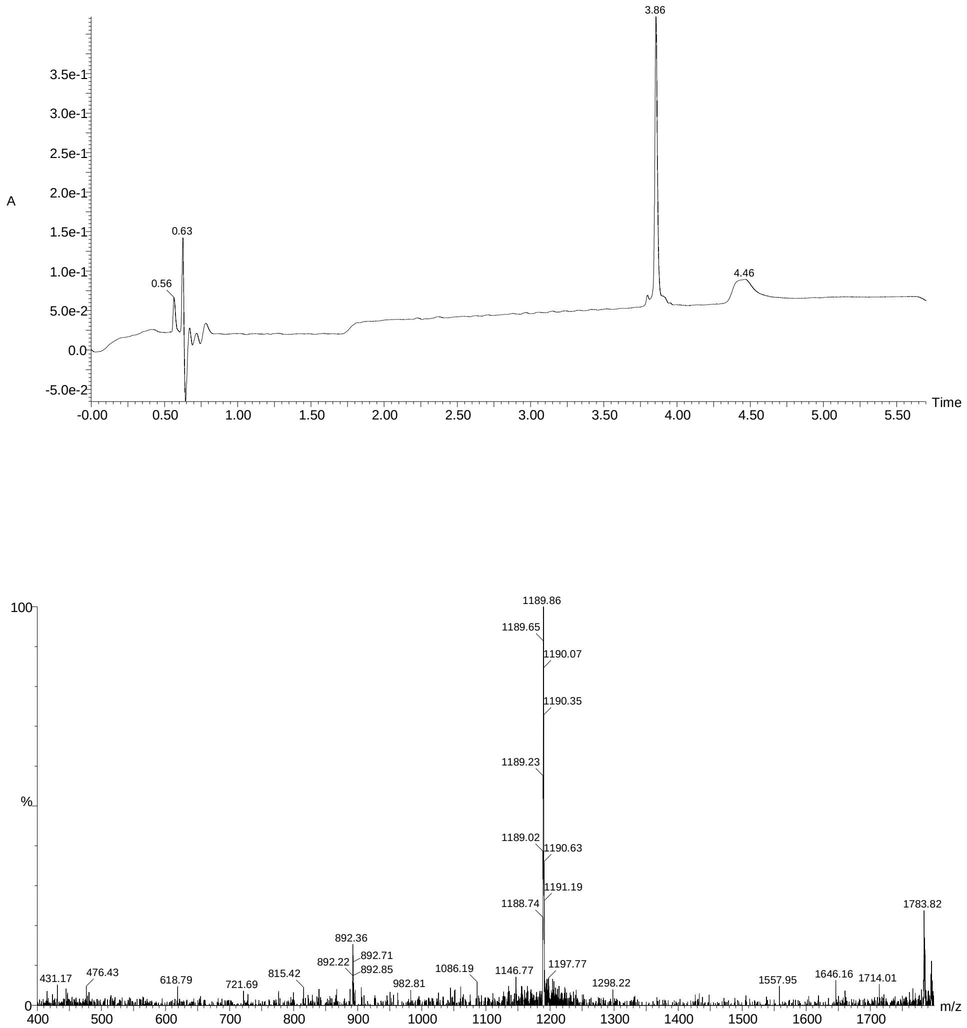

Table2: Peptide 4: upper panel: UPLC-UV Analysis on Waters Acquity BEH C18 column (2.1x 100 mm, 1.7 μm, 130 A), Waters TUV detector λ= 214 nm, solvent A= H2O, 0.1% TFA; B: CH3CN, 0.1% TFA; gradient of B 20%-20% (1')-60% (4'). Lower panel: ESI mass spectrometry on Waters SQ detector: MW theoretical= 3390.72; MW calculated =3390.4

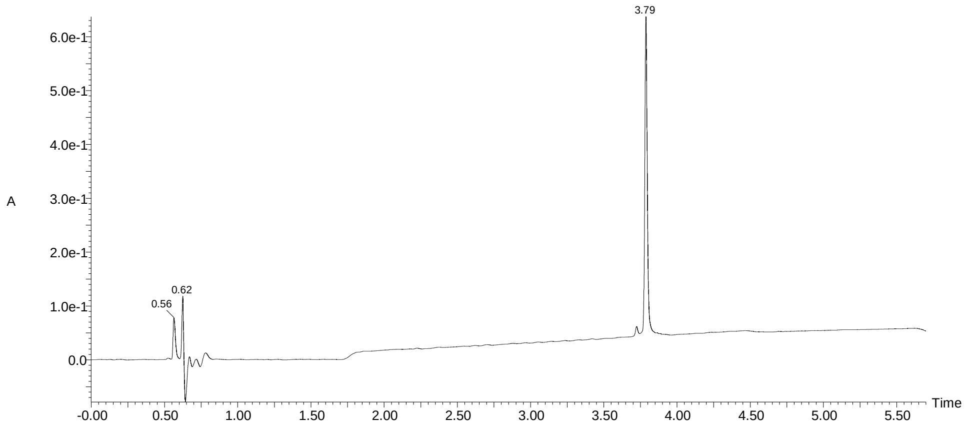

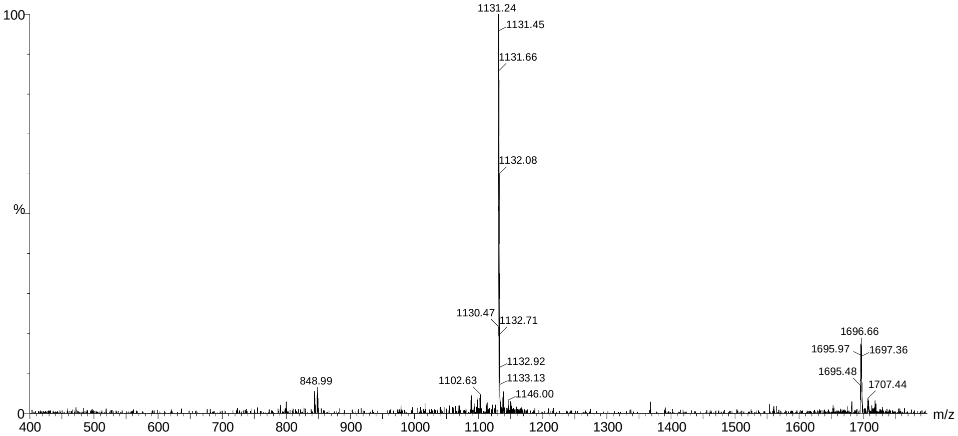

+

Table2: Peptide 5: upper panel: UPLC-UV Analysis on Waters Acquity BEH C18 column (2.1x 100 mm, 1.7 μm, 130 A), Waters TUV detector λ= 214 nm, solvent A= H2O, 0.1% TFA; B: CH3CN, 0.1% TFA; gradient of B 20%-20% (1')-60% (4'). Lower panel: ESI mass spectrometry on Waters SQ detector: MW theoretical= 3386.7; MW calculated = 3386.30

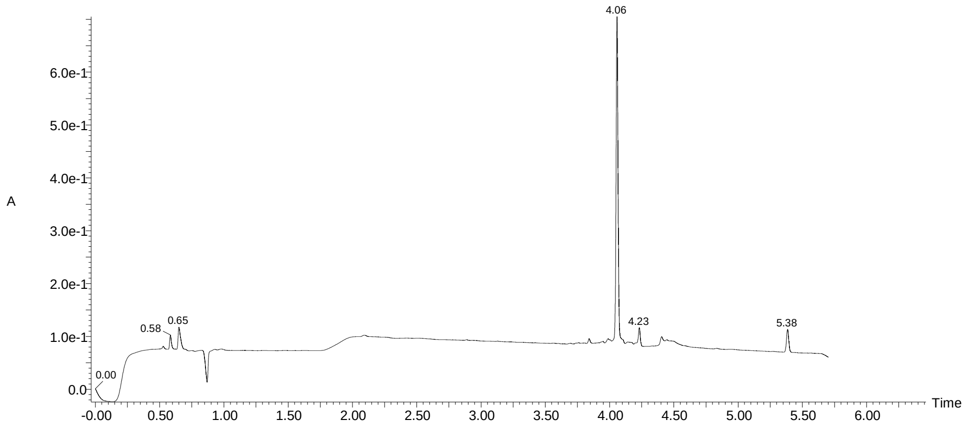

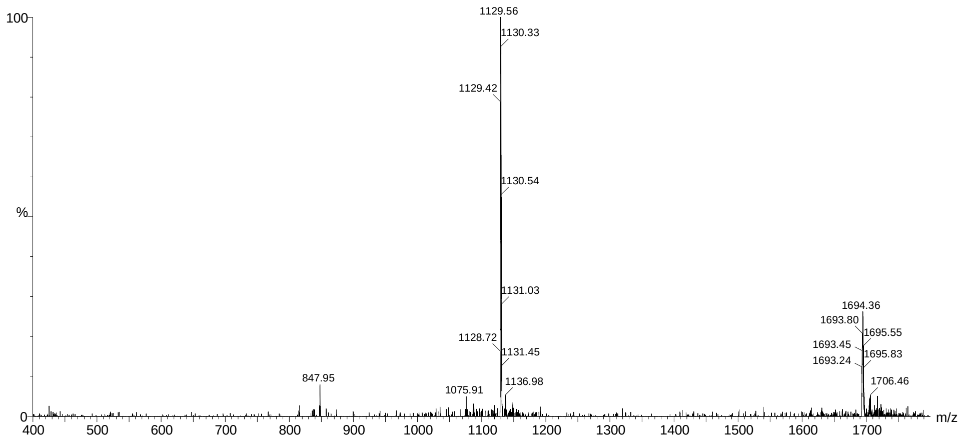

Table2: Peptide 6: upper panel: UPLC-UV Analysis on Waters Acquity BEH C18 column (2.1x 100 mm, 1.7 μm, 130 A), Waters TUV detector λ= 214 nm, solvent A= H2O, 0.1% TFA; B: CH3CN, 0.1% TFA; gradient of B 20%-20% (1')-60% (4'). Lower panel: ESI mass spectrometry on Waters SQ detector: MW theoretical= 3562.91; MW calculated= 3563.22

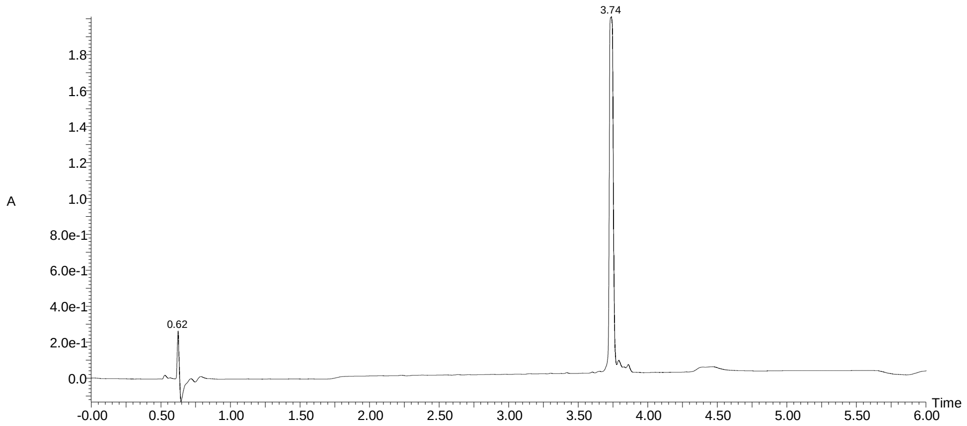

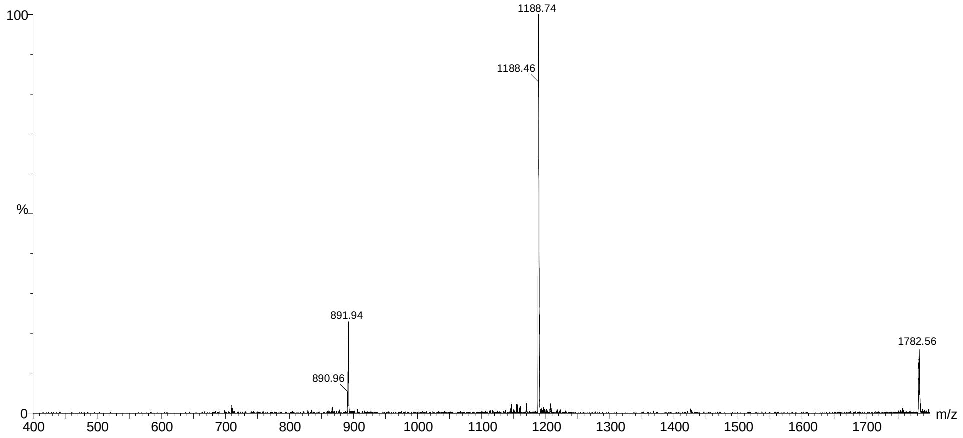

Table2: Peptide 7: upper panel: UPLC-UV Analysis on Waters Acquity BEH C18 column (2.1x 100 mm, 1.7 μm, 130 A), Waters TUV detector λ= 214 nm, solvent A= H2O, 0.1% TFA; B: CH3CN, 0.1% TFA; gradient of B 20%-20% (1')-60% (4'). Lower panel: ESI mass spectrometry on Waters SQ detector: MW theoretical= 3457.88; MW calculated= 3458.5

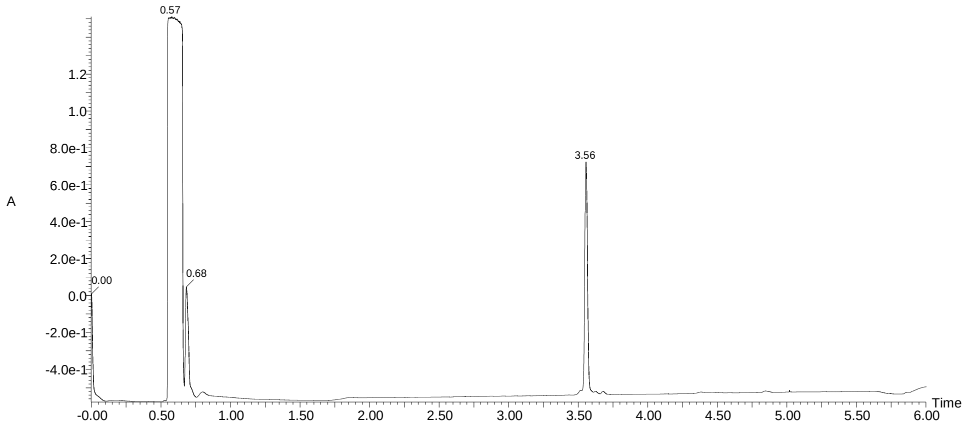

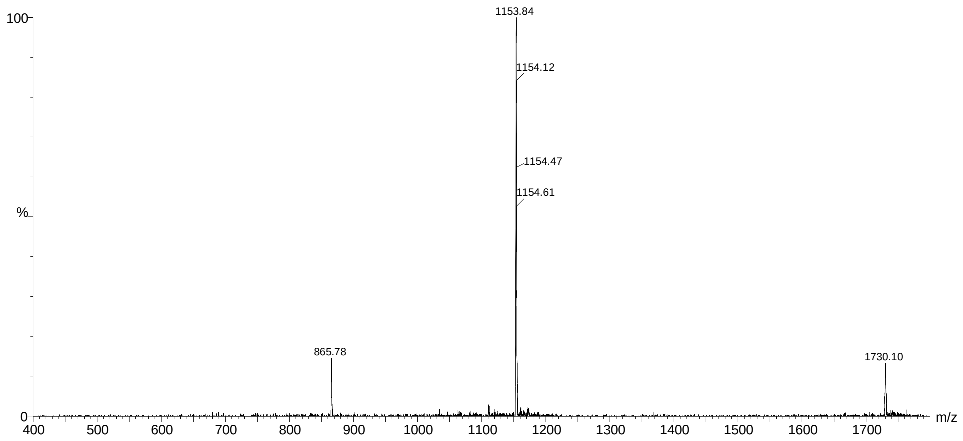

Table2: Peptide 8: upper panel: UPLC-UV Analysis on Waters Acquity BEH C18 column (2.1x 100 mm, 1.7 μm, 130 A), Waters TUV detector λ= 214 nm, solvent A= H2O, 0.1% TFA; B: CH3CN, 0.1% TFA; gradient of B 20%-20% (1')-60% (4'). Lower panel: ESI mass spectrometry on Waters SQ detector: MW theoretical= 3489.86; MW calculated= 3489.5

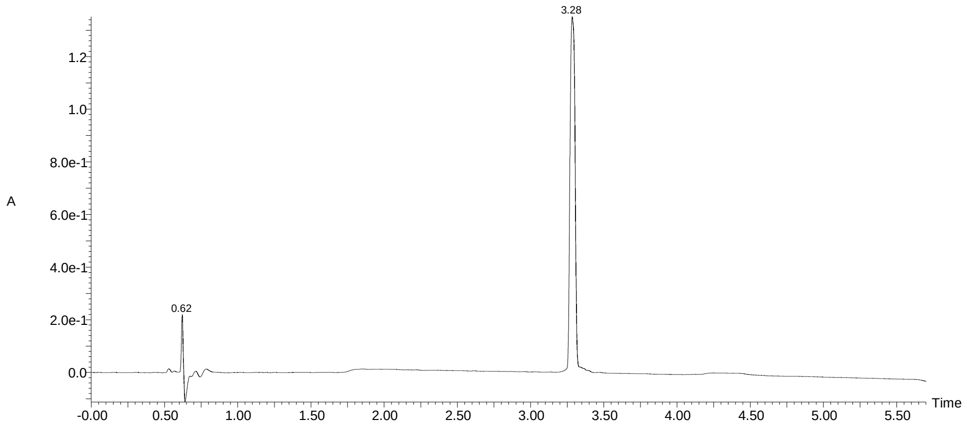

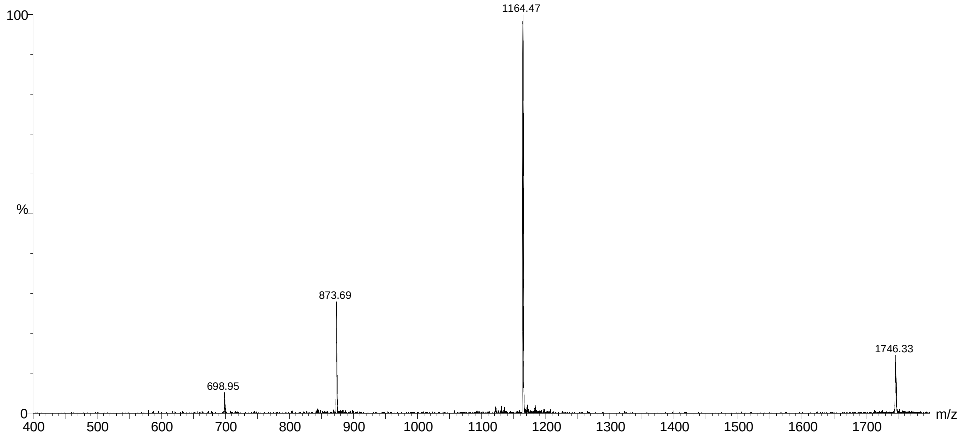

Table2: Peptide 9: upper panel: UPLC-UV Analysis on Waters Acquity BEH C18 column (2.1x 100 mm, 1.7 μm, 130 A), Waters TUV detector λ= 214 nm, solvent A= H2O, 0.1% TFA; B: CH3CN, 0.1% TFA; gradient of B 20%-20% (1')-60% (4'). Lower panel: ESI mass spectrometry on Waters SQ detector: MW theoretical= 3493.87; MW calculated= 3493.3

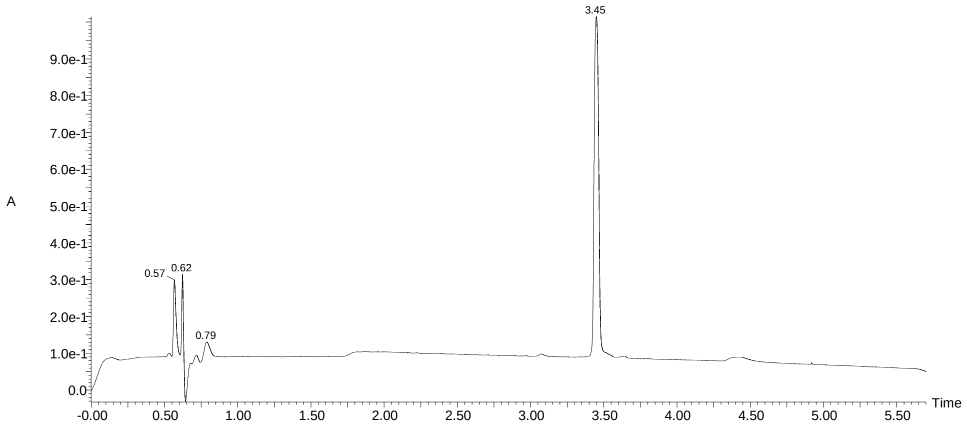

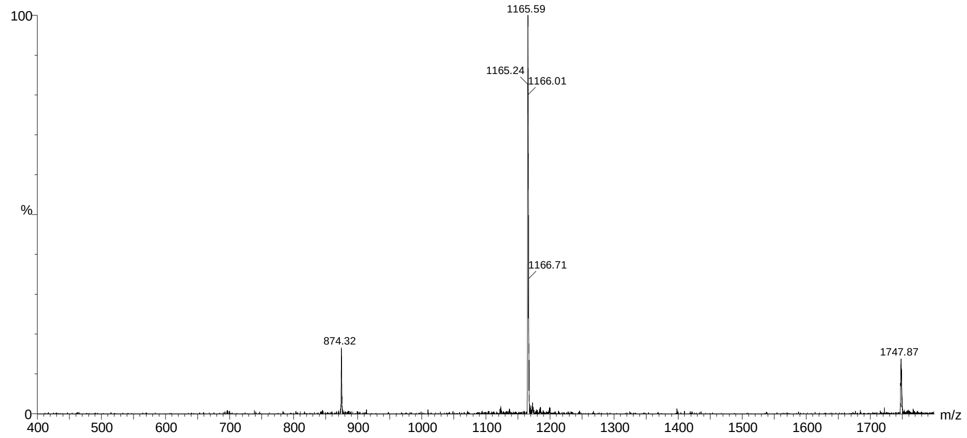

Table2: Peptide 10: upper panel: UPLC-UV Analysis on Waters Acquity BEH C18 column (2.1x 100 mm, 1.7 μm, 130 A), Waters TUV detector λ= 214 nm, solvent A= H2O, 0.1% TFA; B: CH3CN, 0.1% TFA; gradient of B 20%-20% (1')-60% (4'). Lower panel: ESI mass spectrometry on Waters SQ detector: MW theoretical= 3515.8; MW calculated= 3516.0

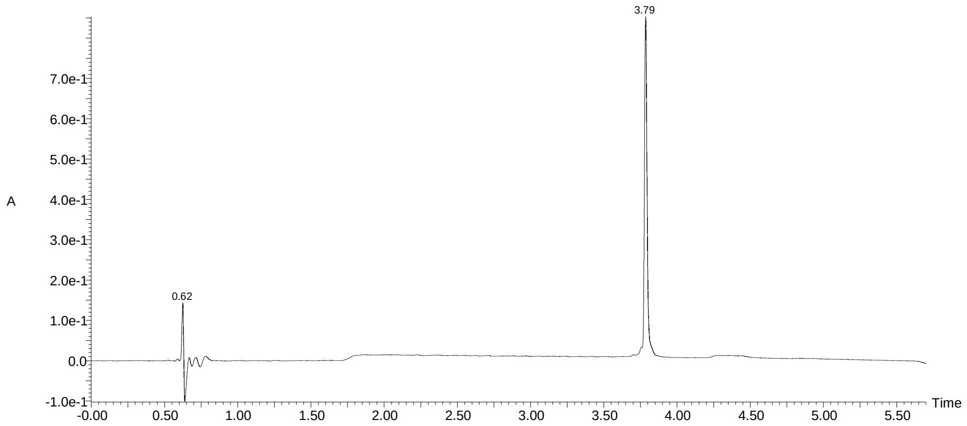

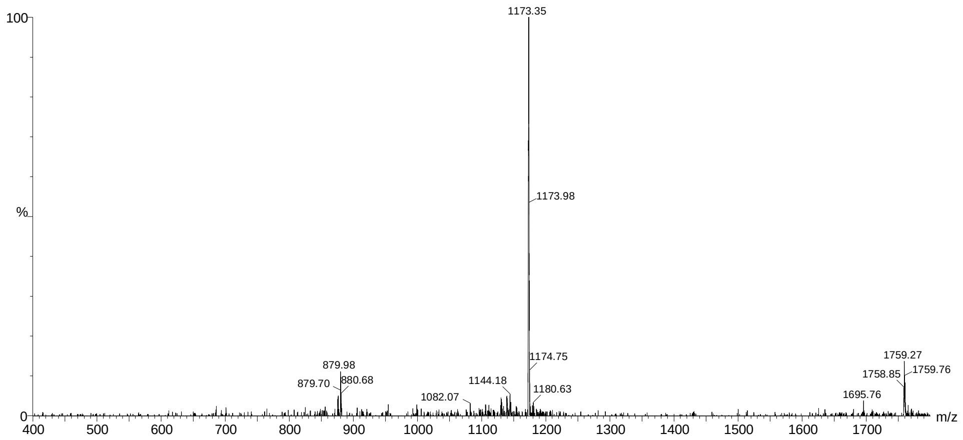

Table2: Peptide 11: upper panel: UPLC-UV Analysis on Waters Acquity BEH C18 column (2.1x 100 mm, 1.7 µm, 130 A), Waters TUV detector λ= 214 nm, solvent A= H2O, 0.1% TFA; B: CH3CN, 0.1% TFA; gradient of B 20%-20% (1')-60% (4'). Lower panel: ESI mass spectrometry on Waters SQ detector: MW theoretical= 3472.74; MW calculated= 3472.9

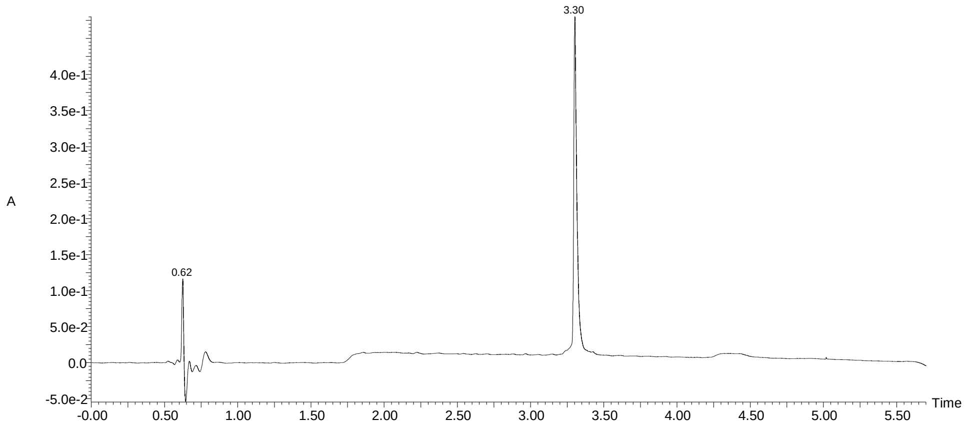

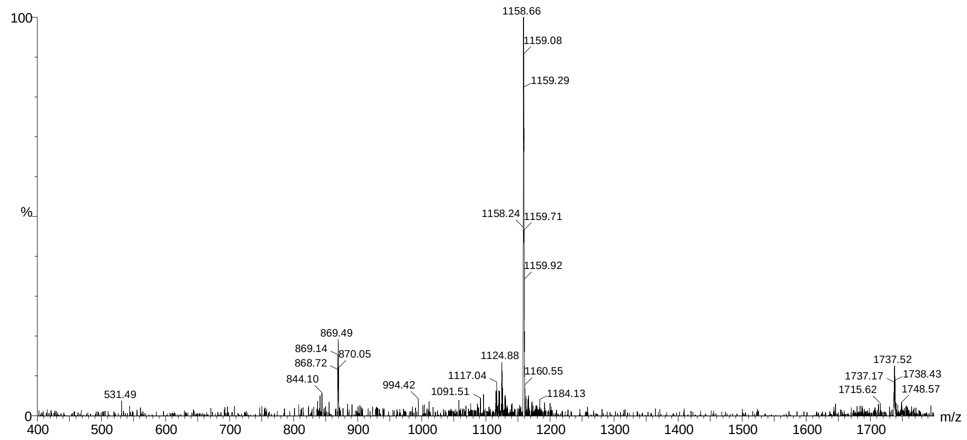

Table2: Peptide 12: upper panel: UPLC-UV Analysis on Waters Acquity BEH C18 column (2.1x 100 mm, 1.7 μm, 130 A), Waters TUV detector λ= 214 nm, solvent A= H2O, 0.1% TFA; B: CH3CN, 0.1% TFA; gradient of B 20%-20% (1')-60% (4'). Lower panel: ESI mass spectrometry on Waters SQ detector: MW theoretical= 3477.78; MW calculated= 3478.86

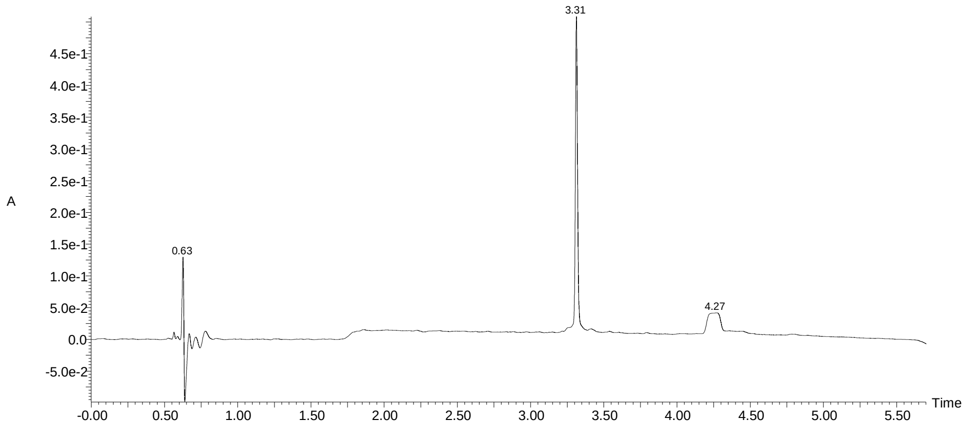

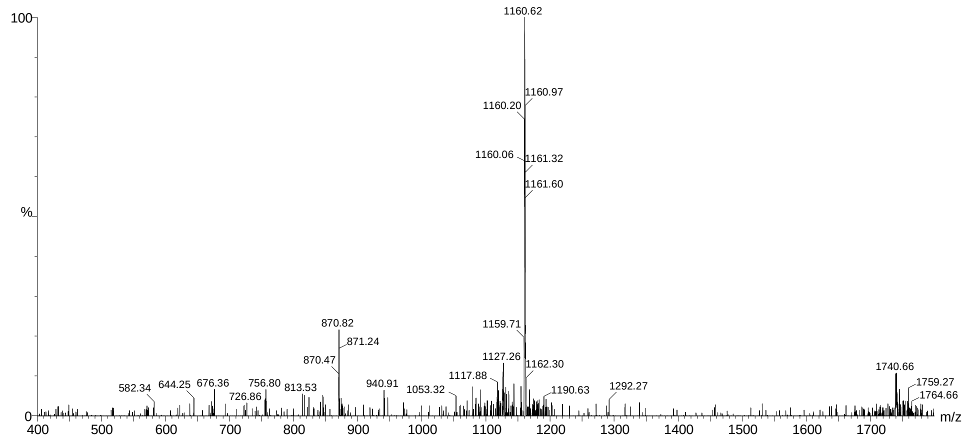

Table2: Peptide 13: upper panel: UPLC-UV Analysis on Waters Acquity BEH C18 column (2.1x 100 mm, 1.7 μm, 130 A), Waters TUV detector λ= 214 nm, solvent A= H2O, 0.1% TFA; B: CH3CN, 0.1% TFA; gradient of B 20%-20% (1')-60% (5')-80% (1'). Lower panel: ESI mass spectrometry on Waters SQ detector: MW theoretical= 3525.8; MW calculated= 3526.5

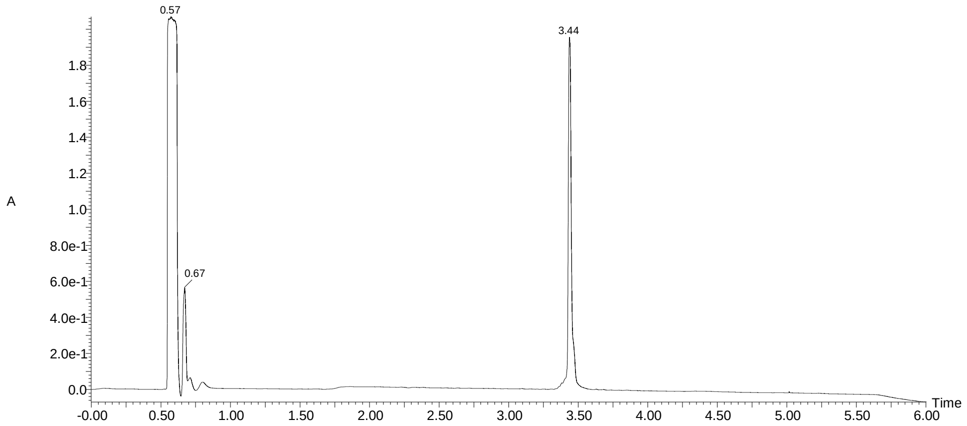

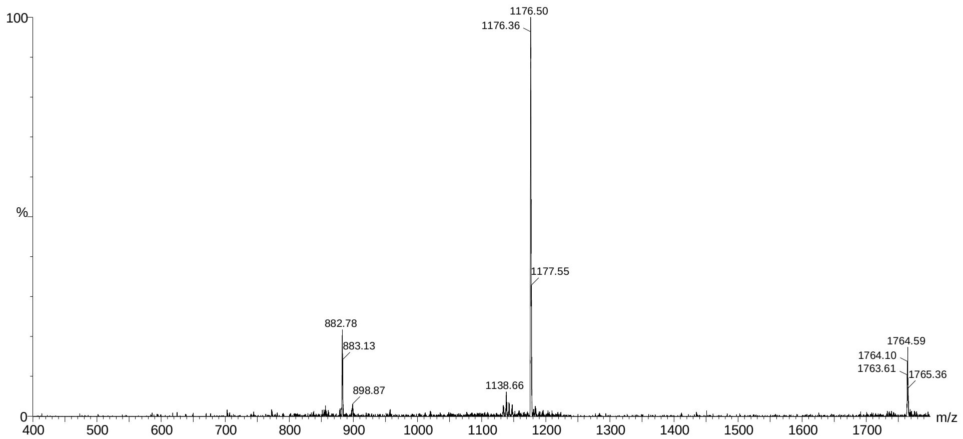

Table2: Peptide 14: upper panel: UPLC-UV Analysis on Waters Acquity BEH C18 column (2.1x 100 mm, 1.7 μm, 130 A), Waters TUV detector λ= 214 nm, solvent A= H2O, 0.1% TFA; B: CH3CN, 0.1% TFA; gradient of B 20%-20% (1')-60% (4'). Lower panel: ESI mass spectrometry on Waters SQ detector: MW theoretical= 3850.2; MW calculated= 3851.60

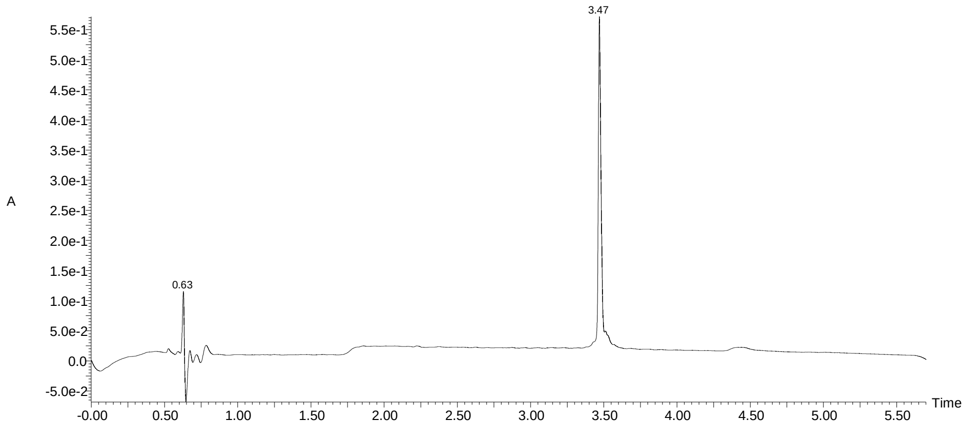

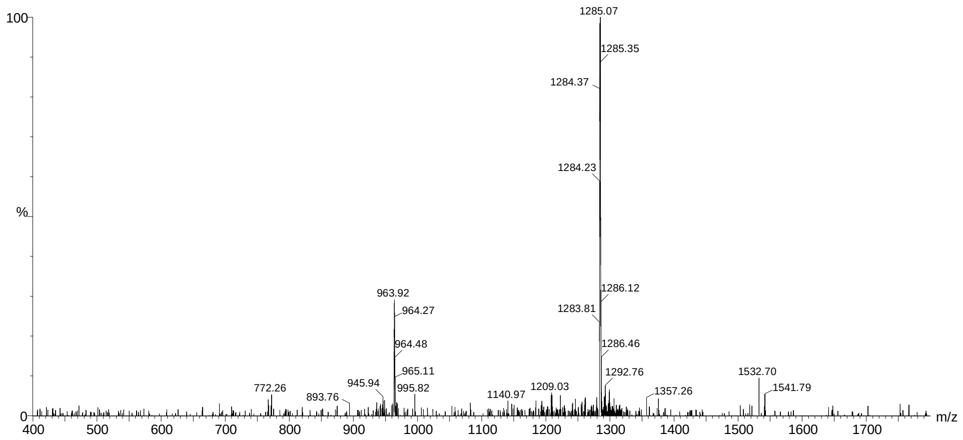

Table2: Peptide 15: upper panel: UPLC-UV Analysis on Waters Acquity BEH C18 column (2.1x 100 mm, 1.7 μm, 130 A), Waters TUV detector λ= 214 nm, solvent A= H2O, 0.1% TFA; B: CH3CN, 0.1% TFA; gradient of B 20%-20% (1')-60% (4'). Lower panel: ESI mass spectrometry on Waters SQ detector: MW theoretical= 3554.02; MW calculated= 3554.82

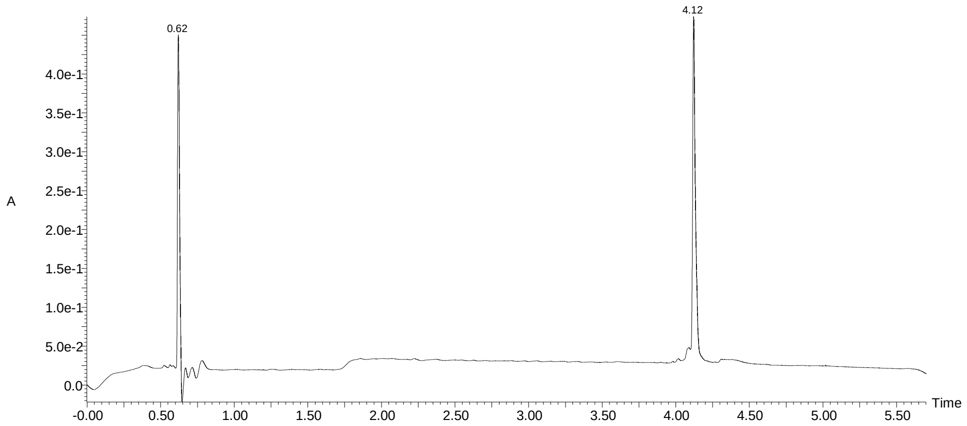

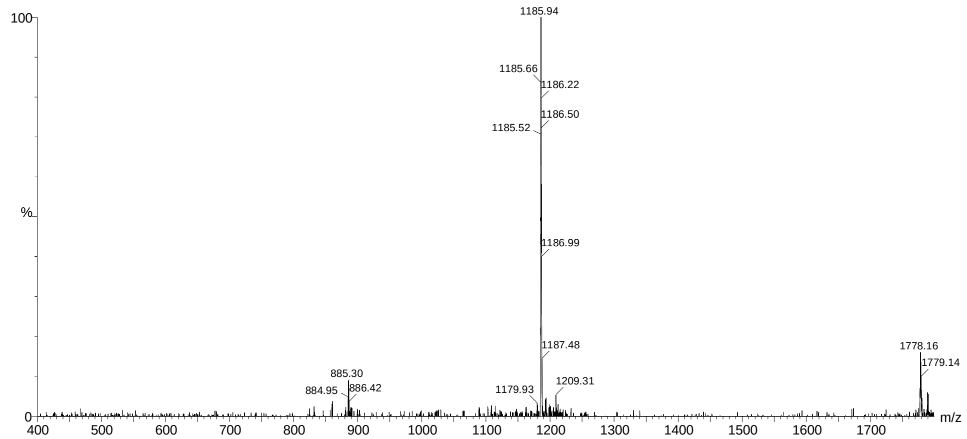

Table2: Peptide 16: upper panel: UPLC-UV Analysis on Waters Acquity BEH C18 column (2.1x 100 mm, 1.7 μm, 130 A), Waters TUV detector λ= 214 nm, solvent A= H2O, 0.1% TFA; B: CH3CN, 0.1% TFA; gradient of B 25%-45% (4'). Lower panel: ESI mass spectrometry on Waters SQ detector: MW theoretical= 3457.83; MW calculated= 3458.16

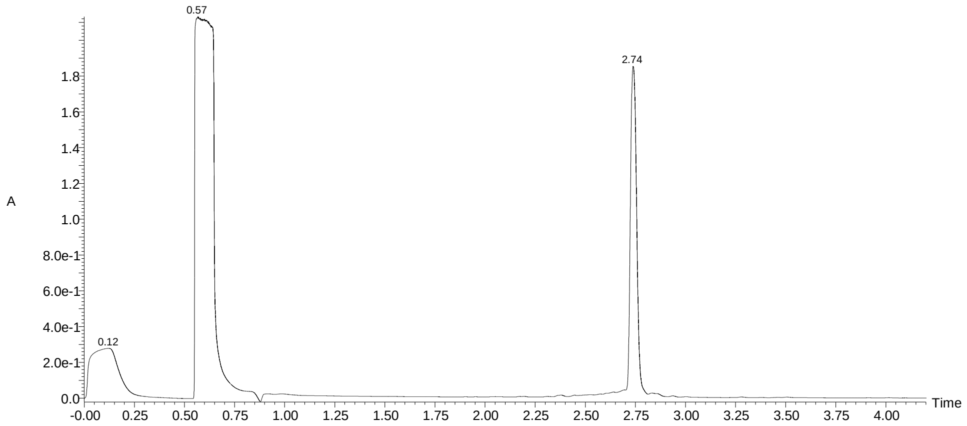

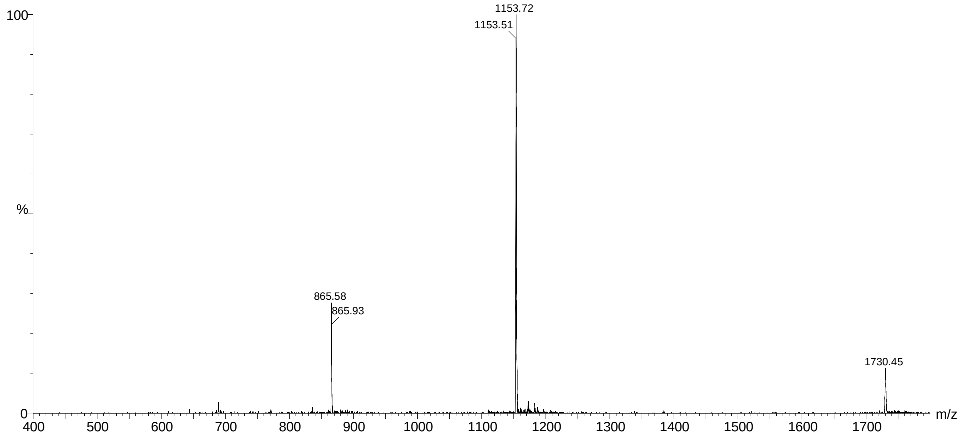

Table2: Peptide 17: upper panel: UPLC-UV Analysis on Waters Acquity BEH C18 column (2.1x 100 mm, 1.7 μm, 130 A), Waters TUV detector λ= 214 nm, solvent A= H2O, 0.1% TFA; B: CH3CN, 0.1% TFA; gradient of B 20%-20% (1')-60% (4'). Lower panel: ESI mass spectrometry on Waters SQ detector: MW theoretical=3655.96; MW calculated = 3656.82

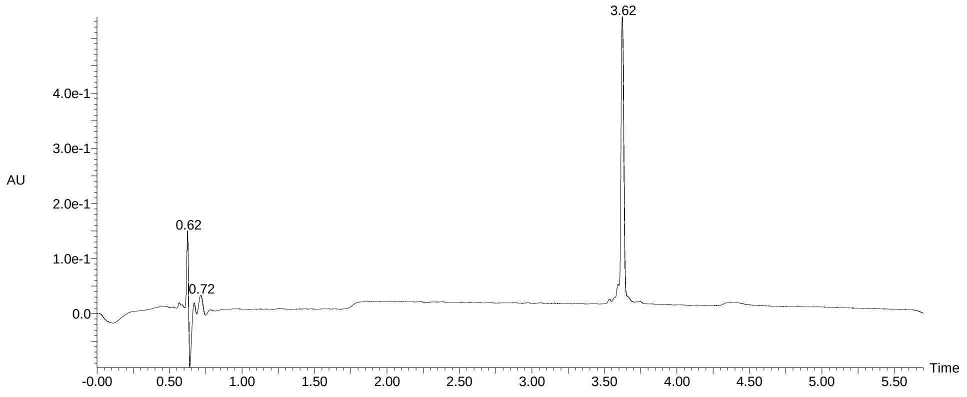

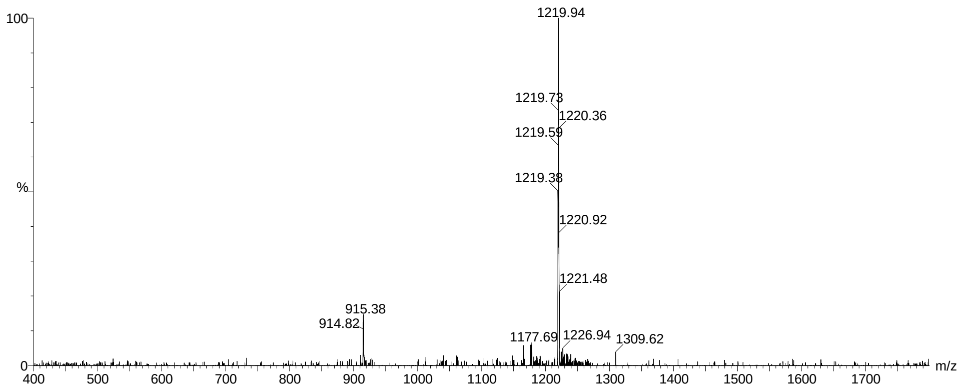

Table2: Peptide 18: upper panel: UPLC-UV Analysis on Waters Acquity BEH C18 column (2.1x 100 mm, 1.7 μm, 130 A), Waters TUV detector λ= 214 nm, solvent A= H2O, 0.1% TFA; B: CH3CN, 0.1% TFA; gradient of B 20%-20% (1')-60% (4'). Lower panel: ESI mass spectrometry on Waters SQ detector: MW theoretical= 3439.72; MW calculated = 3439.80

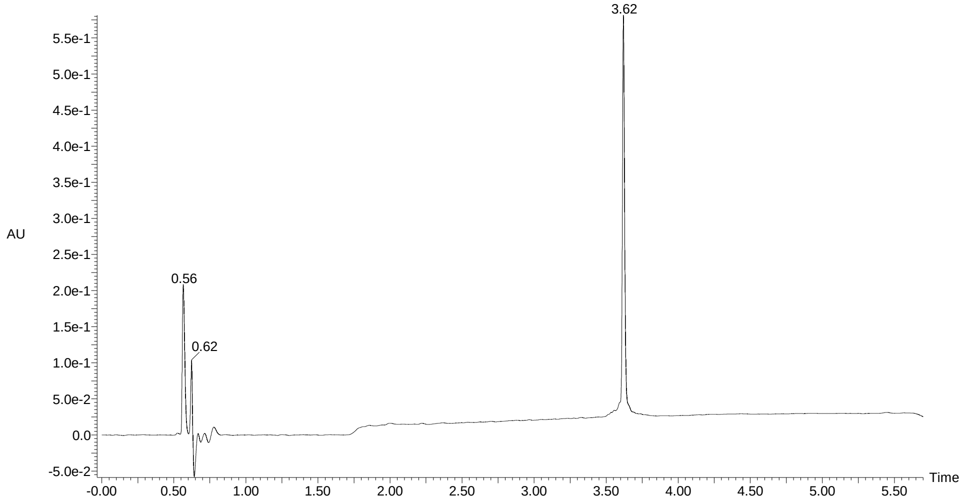

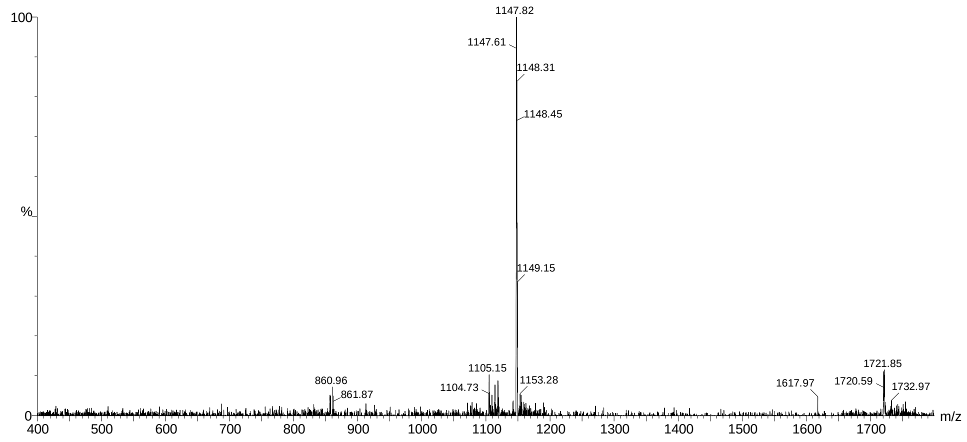

Table2: Peptide 19: upper panel: UPLC-UV Analysis on Waters Acquity BEH C18 column (2.1x 100 mm, 1.7 μm, 130 A), Waters TUV detector λ= 214 nm, solvent A= H2O, 0.1% TFA; B: CH3CN, 0.1% TFA; gradient of B 20%-20% (1')-60% (4'). Lower panel: ESI mass spectrometry on Waters SQ detector: MW theoretical= 3496.68; MW calculated = 3497.34

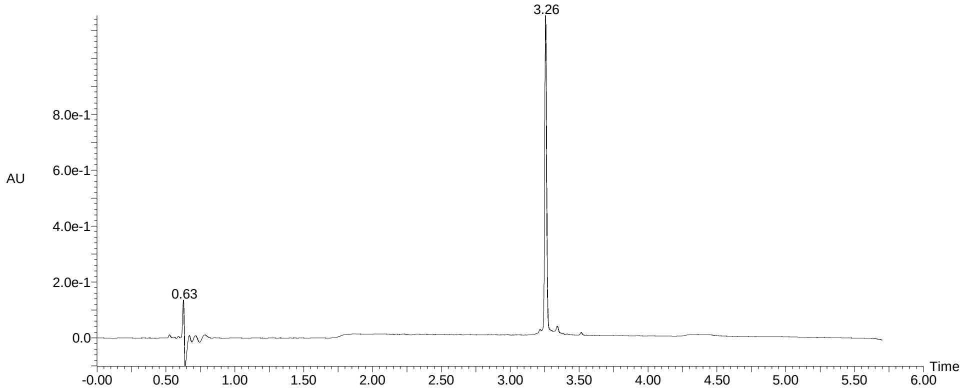

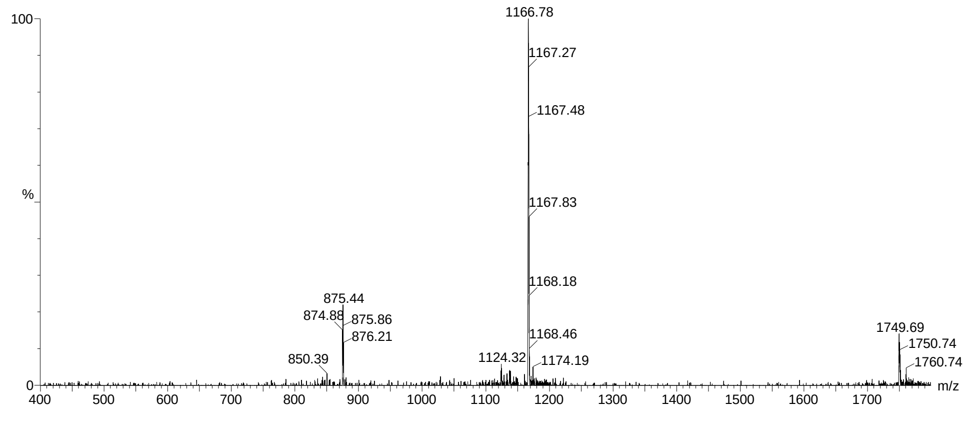

Table2: Peptide 20: upper panel: UPLC-UV Analysis on Waters Acquity BEH C18 column (2.1x 100 mm, 1.7 μm, 130 A), Waters TUV detector λ= 214 nm, solvent A= H2O, 0.1% TFA; B: CH3CN, 0.1% TFA; gradient of B 20%-20% (1')-60% (4'). Lower panel: ESI mass spectrometry on Waters SQ detector: MW theoretical= 3463.73; MW calculated = 3464.4

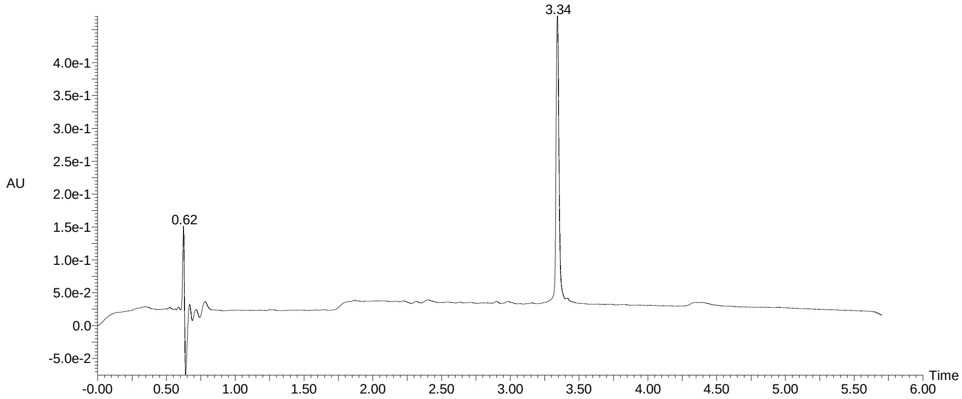

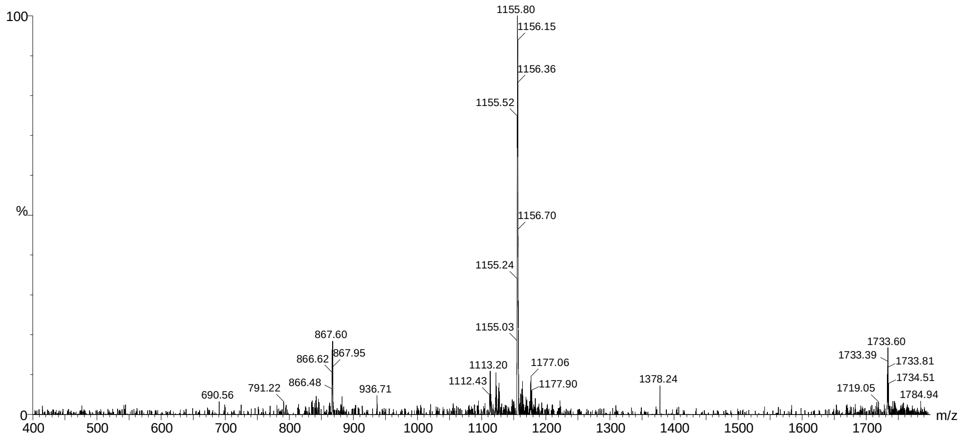

Table2: Peptide 21: upper panel: UPLC-UV Analysis on Waters Acquity BEH C18 column (2.1x 100 mm, 1.7 μm, 130 A), Waters TUV detector λ= 214 nm, solvent A= H2O, 0.1% TFA; B: CH3CN, 0.1% TFA; gradient of B 20%-20% (1')-60% (4'). Lower panel: ESI mass spectrometry on Waters SQ detector: MW theoretical= 3717.18; MW calculated = 3716. 60

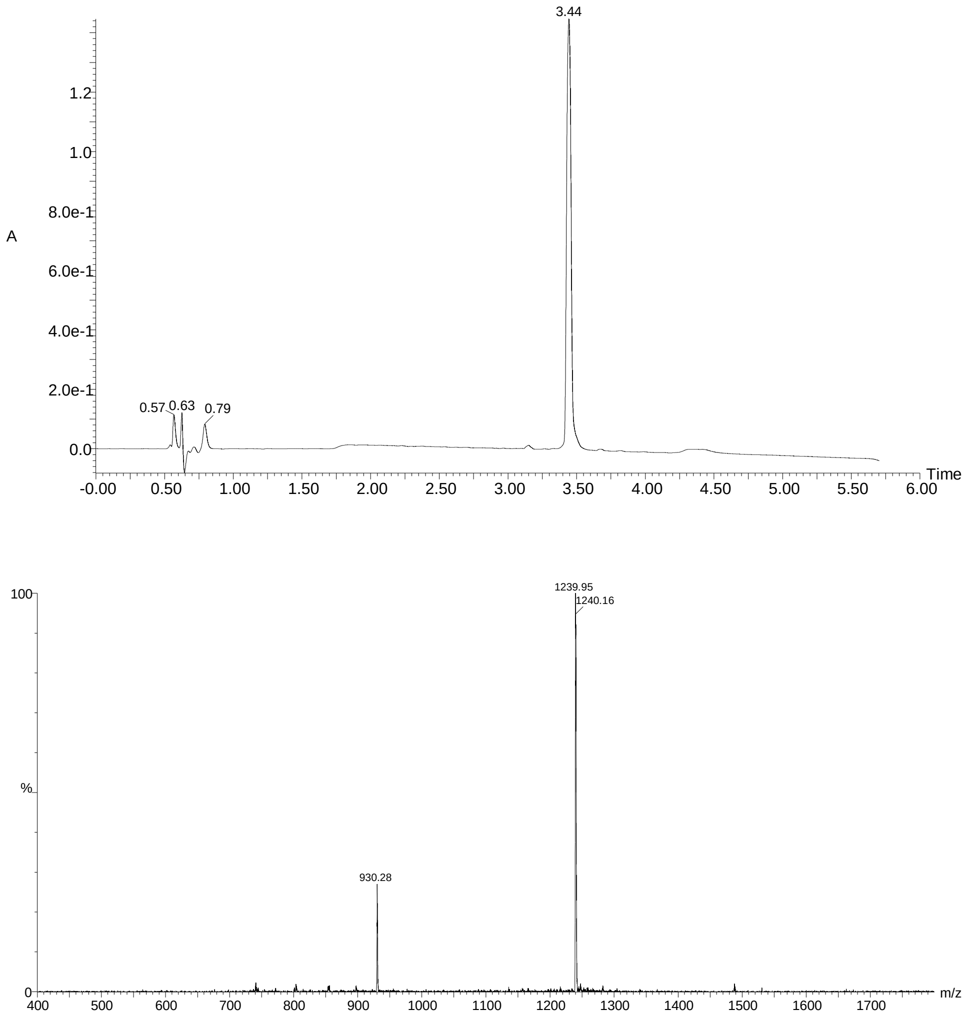

Table2: Peptide 22: upper panel: UPLC-UV Analysis on Waters Acquity BEH C18 column (2.1x 100 mm, 1.7 μm, 130 A), Waters TUV detector λ= 214 nm, solvent A= H2O, 0.1% TFA; B: CH3CN, 0.1% TFA; gradient of B 20%-20% (1')-60% (4'). Lower panel: ESI mass spectrometry on Waters SQ detector: MW theoretical= 3559.89; MW calculated = 3558.7

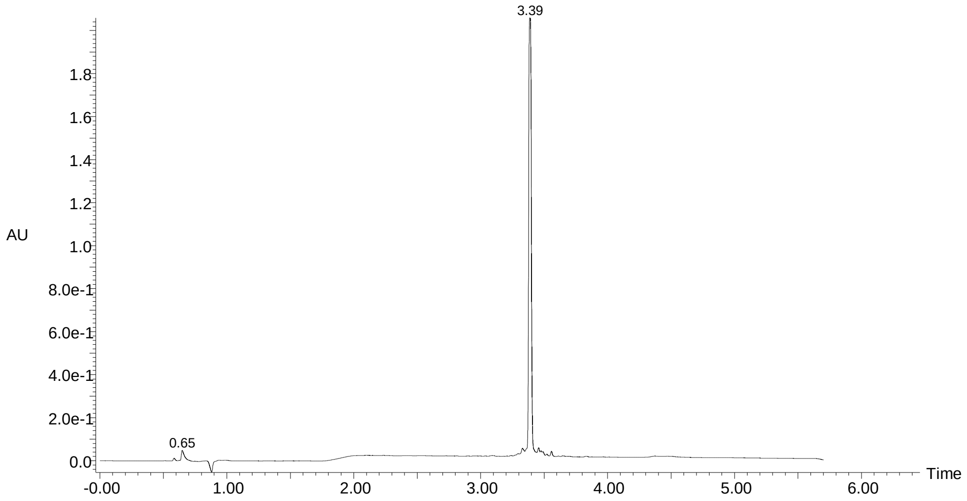

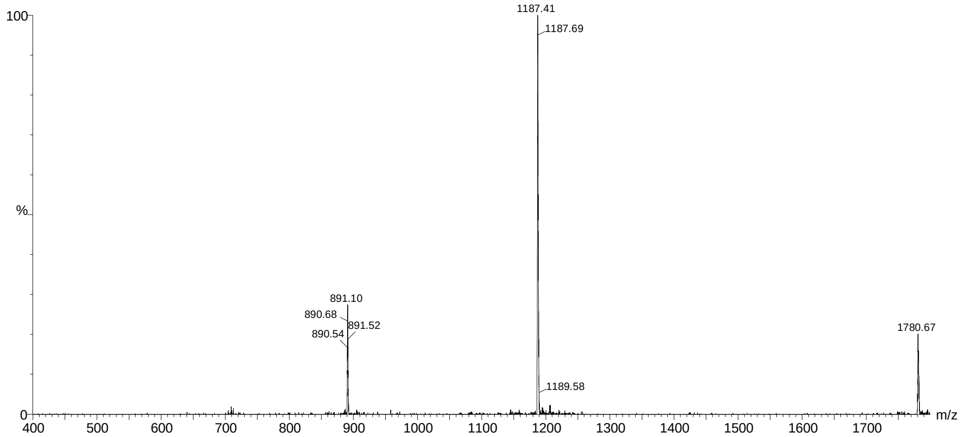

Table2: Peptide 23: upper panel: UPLC-UV Analysis on Waters Acquity BEH C18 column (2.1x 100 mm, 1.7 μm, 130 A), Waters TUV detector λ= 214 nm, solvent A= H2O, 0.1% TFA; B: CH3CN, 0.1% TFA; gradient of B 20%-20% (1')-60% (4'). Lower panel: ESI mass spectrometry on Waters SQ detector: MW theoretical= 3577.89; MW calculated = 3557.3

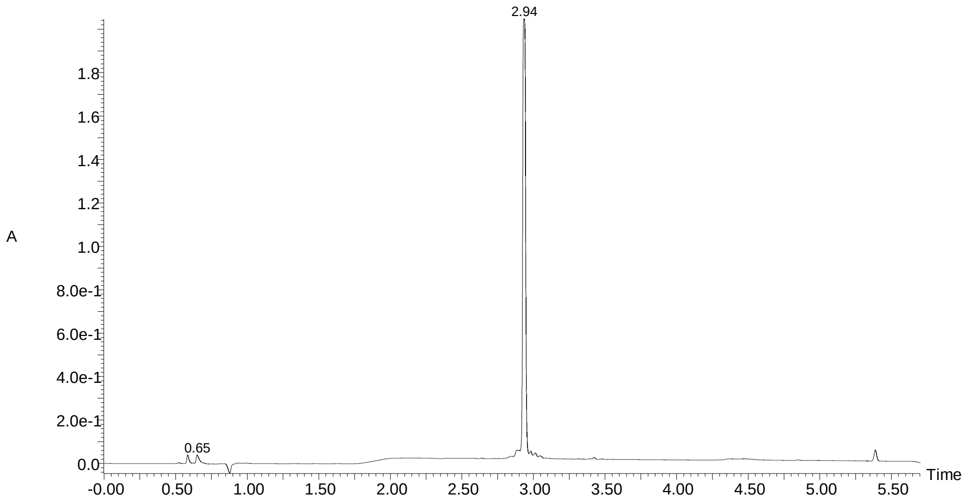

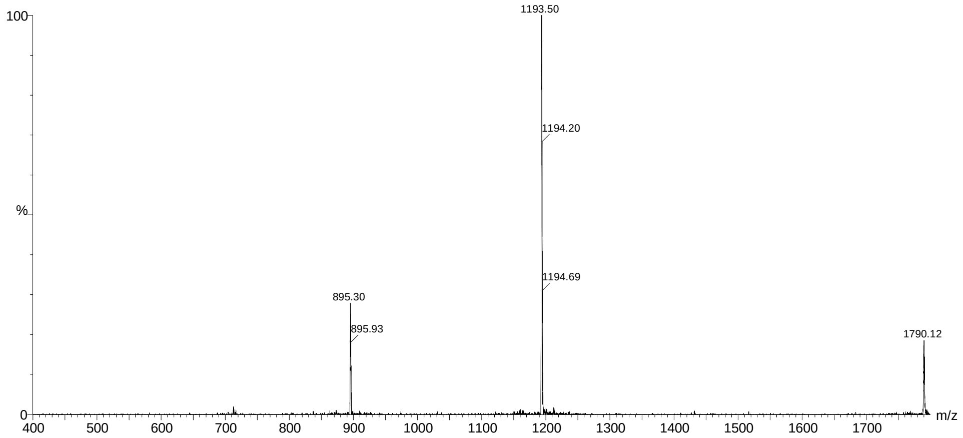

Table2: Peptide 24: upper panel: UPLC-UV Analysis on Waters Acquity BEH C18 column (2.1x 100 mm, 1.7 μm, 130 A), Waters TUV detector λ= 214 nm, solvent A= H2O, 0.1% TFA; B: CH3CN, 0.1% TFA; gradient of B 20%-20% (1')-60% (4'). Lower panel: ESI mass spectrometry on Waters SQ detector: MW theoretical= 3444.78; MW calculated = 3444.2

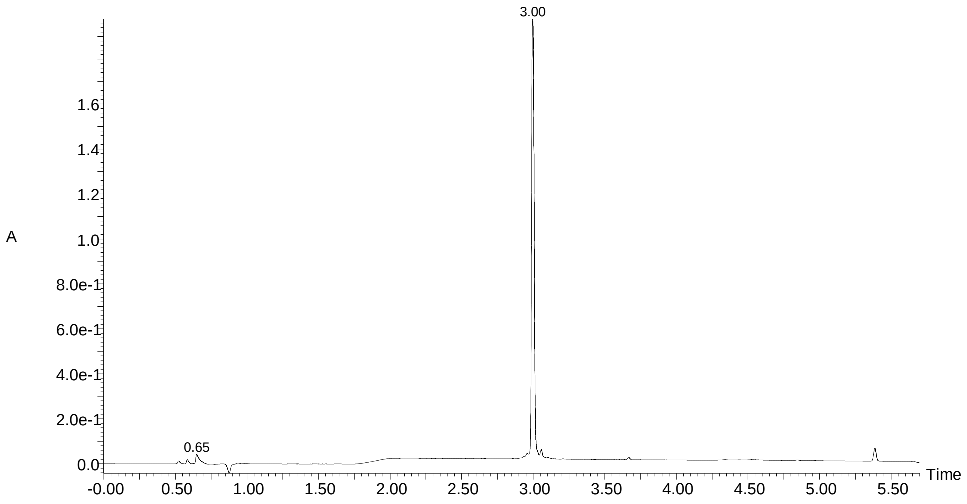

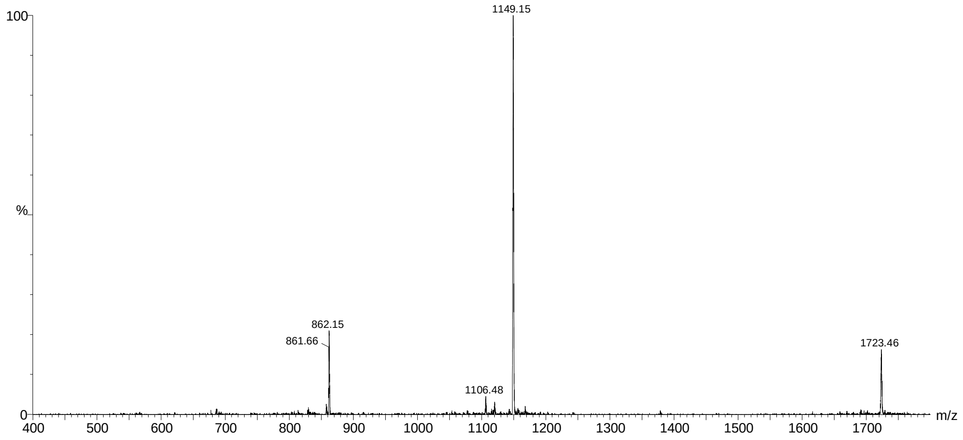

Table2: Peptide 25: upper panel: UPLC-UV Analysis on Waters Acquity BEH C18 column (2.1x 100 mm, 1.7 μm, 130 A), Waters TUV detector λ= 214 nm, solvent A= H2O, 0.1% TFA; B: CH3CN, 0.1% TFA; gradient of B 20%-20% (1')-60% (4'). Lower panel: ESI mass spectrometry on Waters SQ detector: MW theoretical= 3482.8; MW calculated = 3482.04

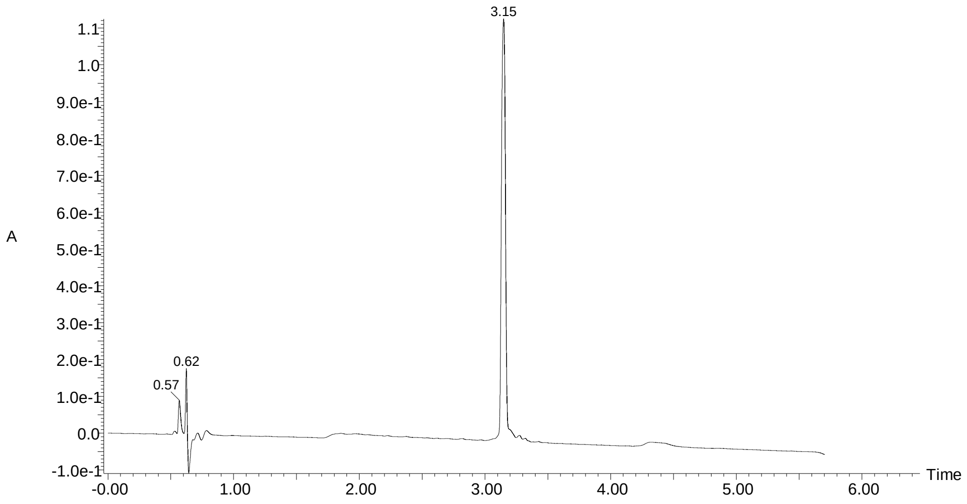

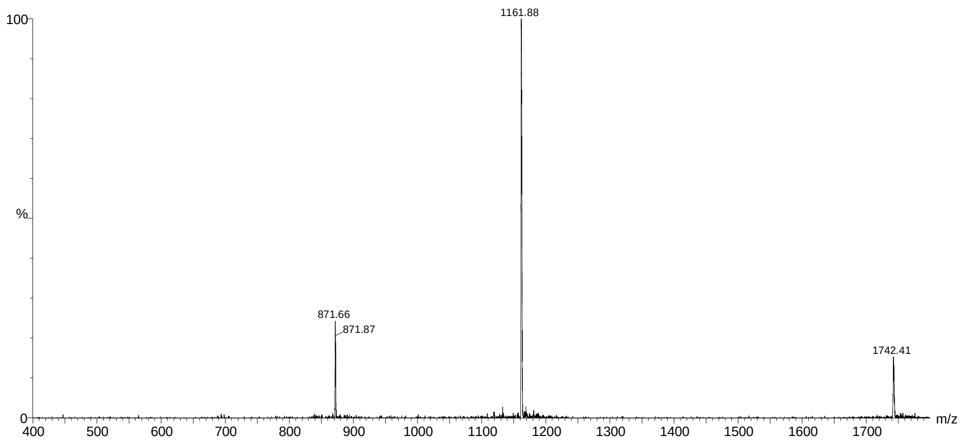

Table2: Peptide 26: upper panel: UPLC-UV Analysis on Waters Acquity BEH C18 column (2.1x 100 mm, 1.7 μm, 130 A), Waters TUV detector λ= 214 nm, solvent A= H2O, 0.1% TFA; B: CH3CN, 0.1% TFA; gradient of B 20%-20% (1')-60% (4'). Lower panel: ESI mass spectrometry on Waters SQ detector: MW theoretical= 3373.78; MW calculated = 3373.53

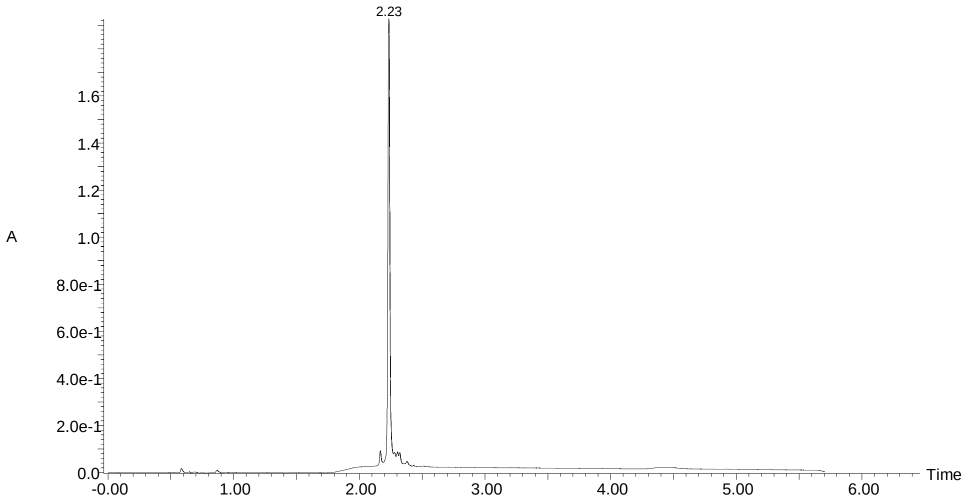

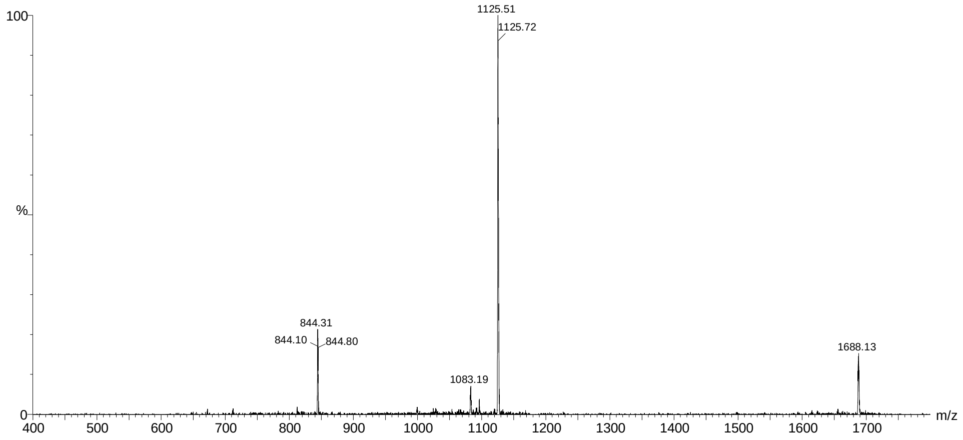

Table2: Peptide 27: upper panel: UPLC-UV Analysis on Waters Acquity BEH C18 column (2.1x 100 mm, 1.7 μm, 130 A), Waters TUV detector λ= 214 nm, solvent A= H2O, 0.1% TFA; B: CH3CN, 0.1% TFA; gradient of B 20%-20% (1')-60% (4'). Lower panel: ESI mass spectrometry on Waters SQ detector: MW theoretical= 3495.75; MW calculated = 3495.66

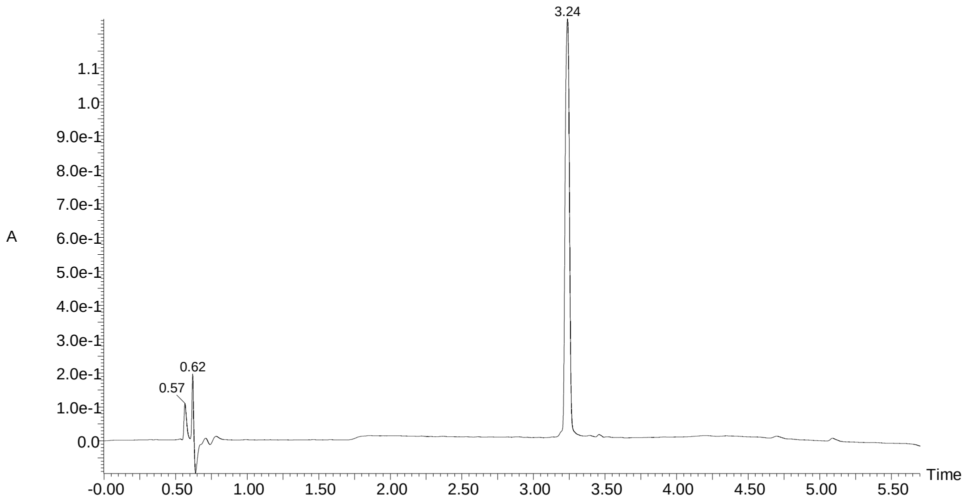

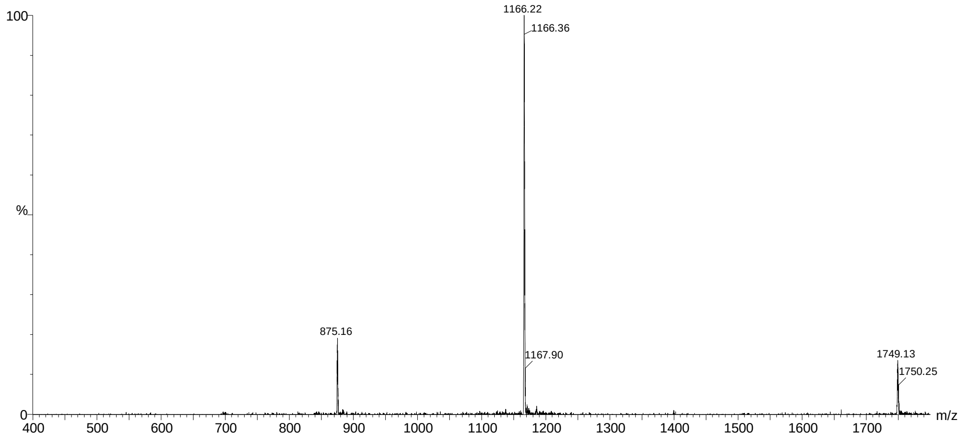

Table2: Peptide 28: upper panel: UPLC-UV Analysis on Waters Acquity BEH C18 column (2.1x 100 mm, 1.7 μm, 130 A), Waters TUV detector λ= 214 nm, solvent A= H2O, 0.1% TFA; B: CH3CN, 0.1% TFA; gradient of B 20%-20% (1')-60% (4'). Lower panel: ESI mass spectrometry on Waters SQ detector: MW theoretical= 3436.66; MW calculated = 3437.1

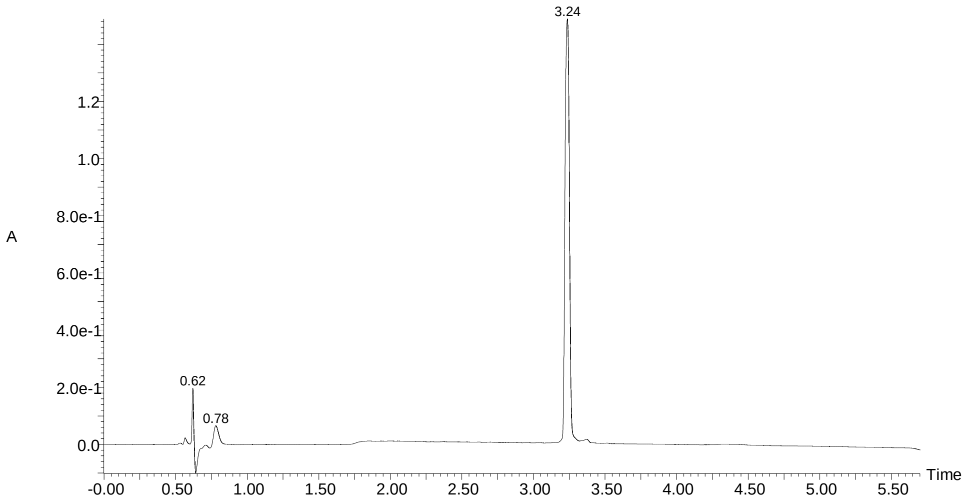

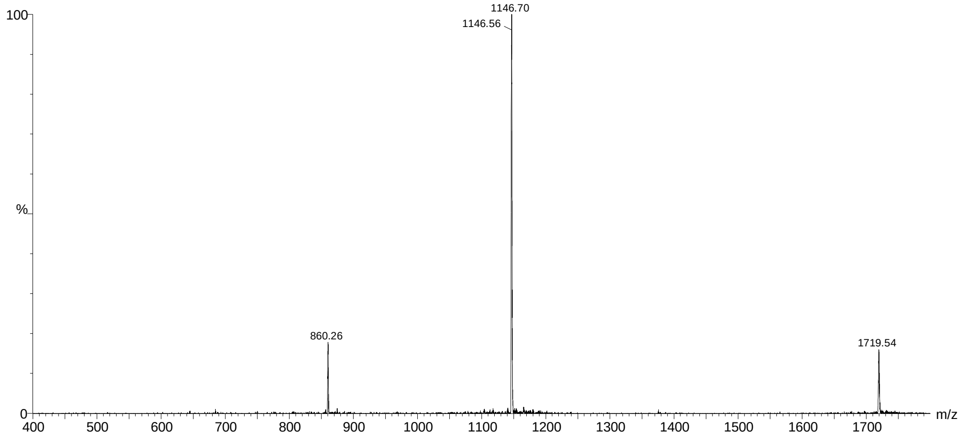

Table2: Peptide 29: upper panel: UPLC-UV Analysis on Waters Acquity BEH C18 column (2.1x 100 mm, 1.7 μm, 130 A), Waters TUV detector λ= 214 nm, solvent A= H2O, 0.1% TFA; B: CH3CN, 0.1% TFA; gradient of B 20%-20% (1')-60% (4'). Lower panel: ESI mass spectrometry on Waters SQ detector: MW theoretical= 3477.78; MW calculated = 3478.44

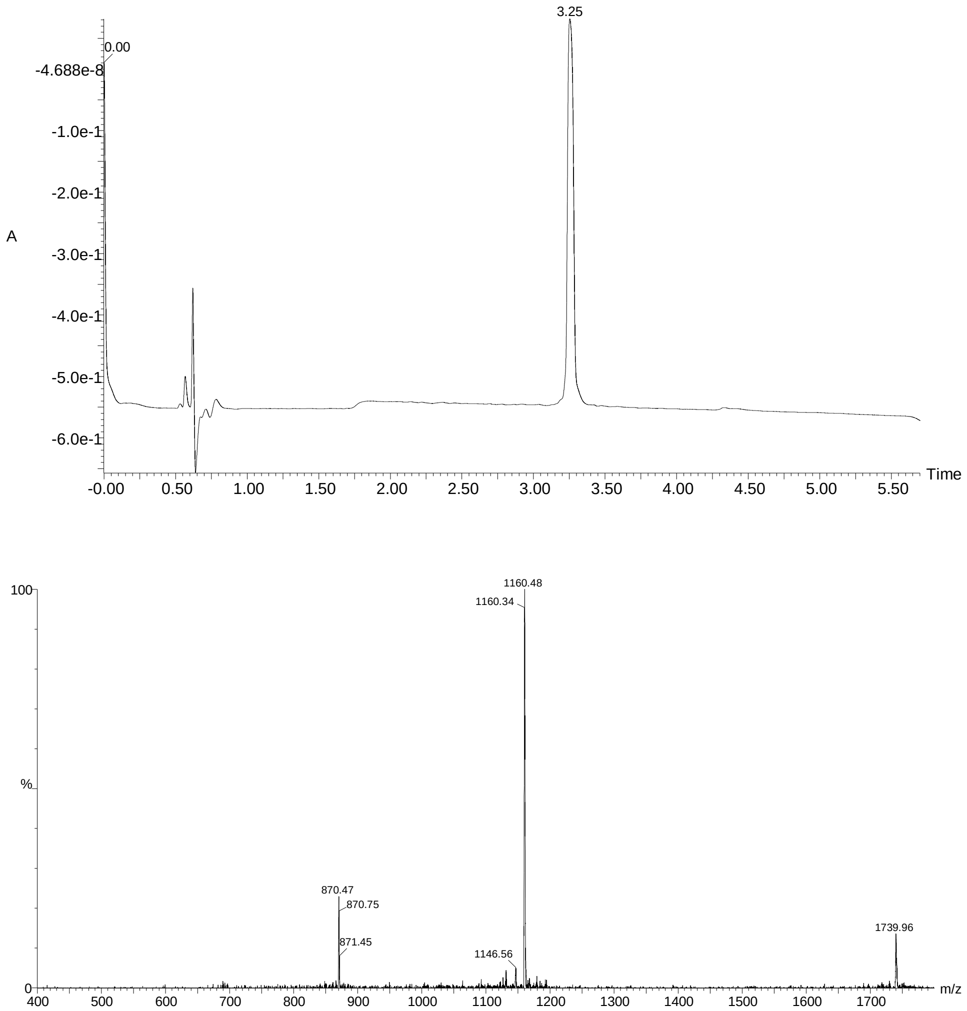

Table2: Peptide 30: upper panel: UPLC-UV Analysis on Waters Acquity BEH C18 column (2.1x 100 mm, 1.7 μm, 130 A), Waters TUV detector λ= 214 nm, solvent A= H2O, 0.1% TFA; B: CH3CN, 0.1% TFA; gradient of B 20%-20% (1')-60% (4'). Lower panel: ESI mass spectrometry on Waters SQ detector: MW theoretical= 3539.89; MW calculated = 3540.99

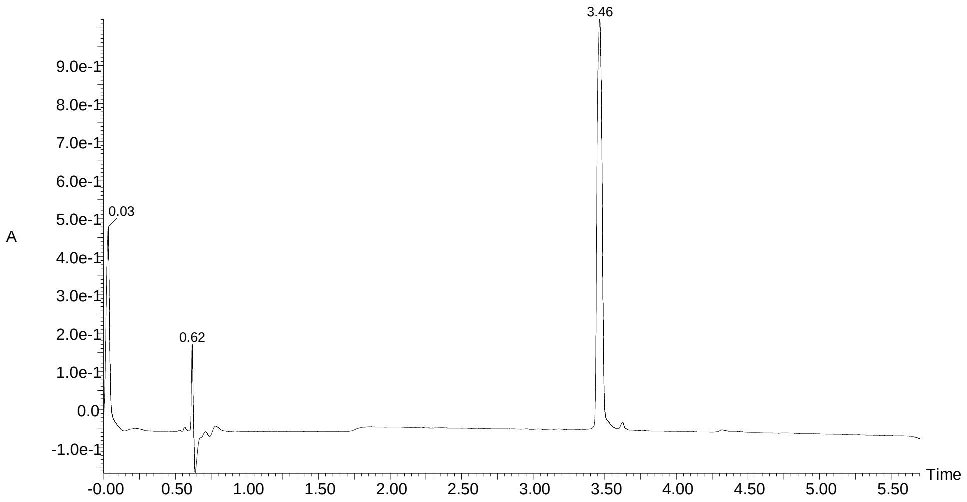

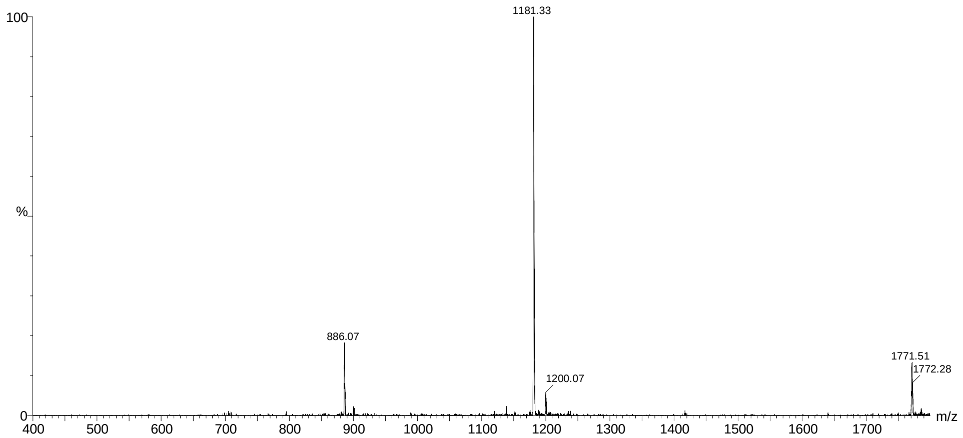

Table2: Peptide 31: upper panel: UPLC-UV Analysis on Waters Acquity BEH C18 column (2.1x 100 mm, 1.7 μm, 130 A), Waters TUV detector λ= 214 nm, solvent A= H2O, 0.1% TFA; B: CH3CN, 0.1% TFA; gradient of B 20%-20% (1')-60% (4'). Lower panel: ESI mass spectrometry on Waters SQ detector: MW theoretical= 3539.80; MW calculated = 3539.94

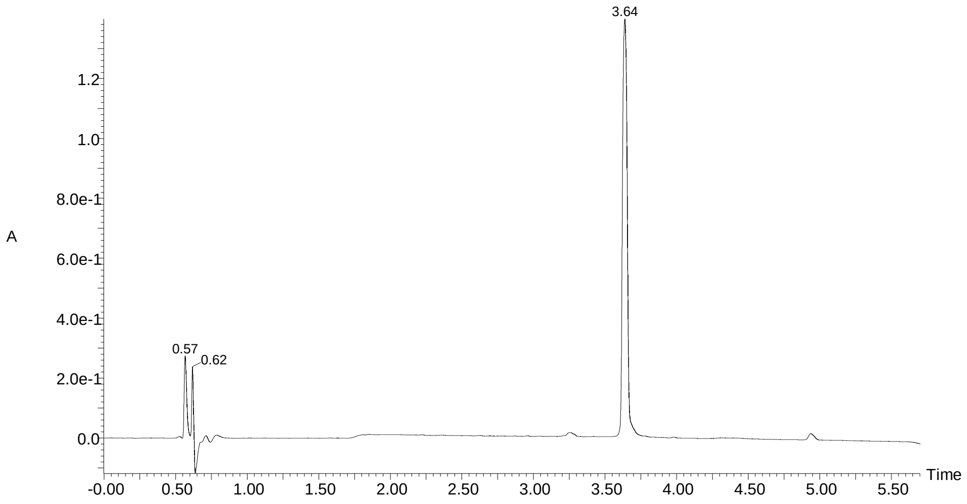

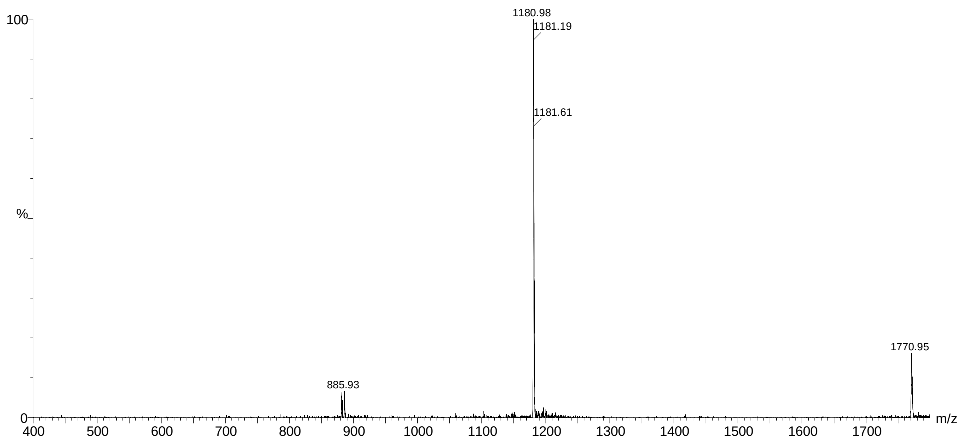

Table2: Peptide 32: upper panel: UPLC-UV Analysis on Waters Acquity BEH C18 column (2.1x 100 mm, 1.7 μm, 130 A), Waters TUV detector λ= 214 nm, solvent A= H2O, 0.1% TFA; B: CH3CN, 0.1% TFA; gradient of B 20%-20% (1')-60% (4'). Lower panel: ESI mass spectrometry on Waters SQ detector: MW theoretical= 3506.84; MW calculated = 3507.42

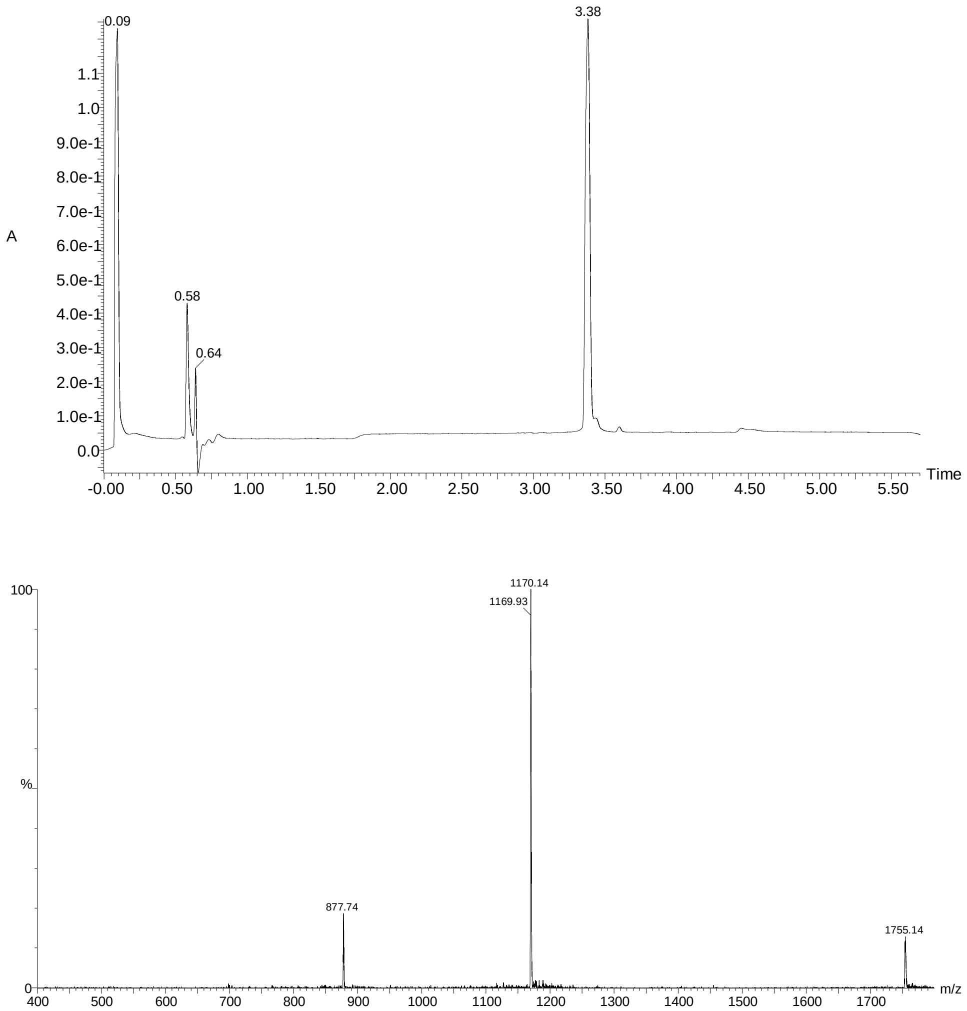

Table2: Peptide 33: upper panel: UPLC-UV Analysis on Waters Acquity BEH C18 column (2.1x 100 mm, 1.7 μm, 130 A), Waters TUV detector λ= 214 nm, solvent A= H2O, 0.1% TFA; B: CH3CN, 0.1% TFA; gradient of B 20%-20% (1')-60% (4'). Lower panel: ESI mass spectrometry on Waters SQ detector: MW theoretical= 3491.86; MW calculated = 3491.25

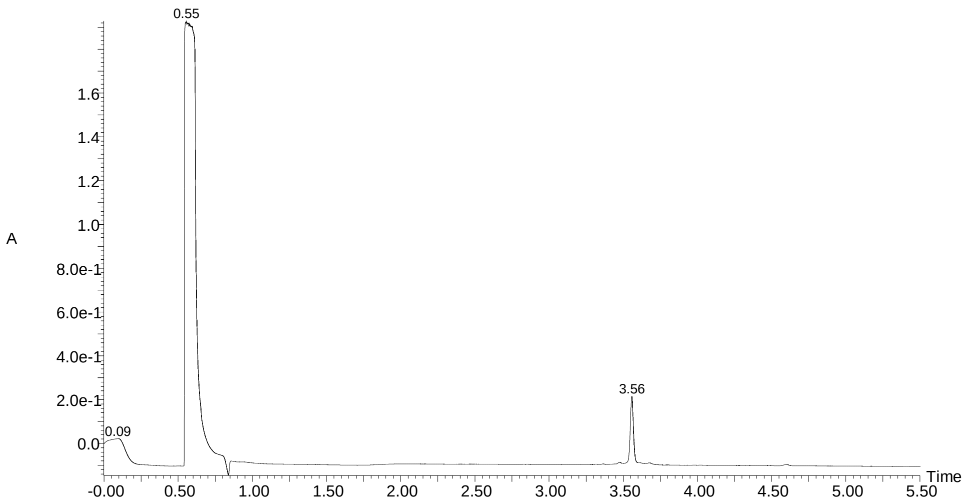

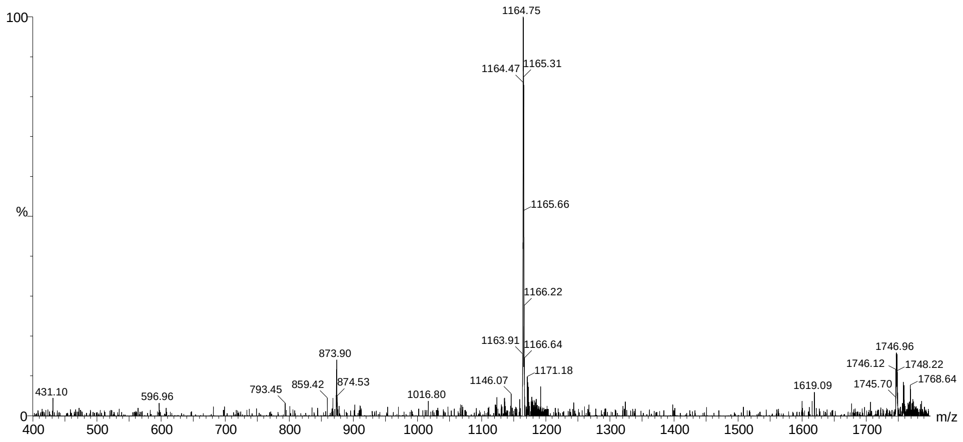

Table2: Peptide 34: upper panel: UPLC-UV Analysis on Waters Acquity BEH C18 column (2.1x 100 mm, 1.7 μm, 130 A), Waters TUV detector λ= 214 nm, solvent A= H2O, 0.1% TFA; B: CH3CN, 0.1% TFA; gradient of B 20%-20% (1')-60% (4'). Lower panel: ESI mass spectrometry on Waters SQ detector: MW theoretical= 3524.81; MW calculated = 3524.61

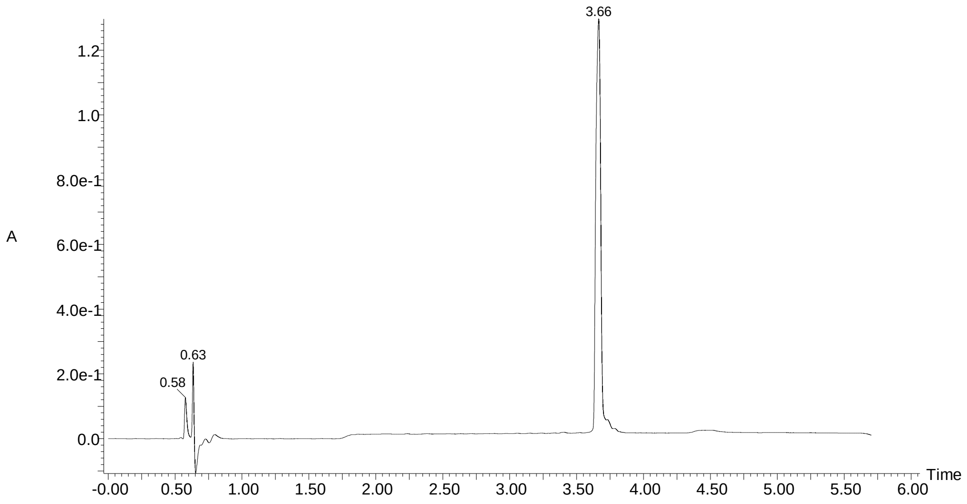

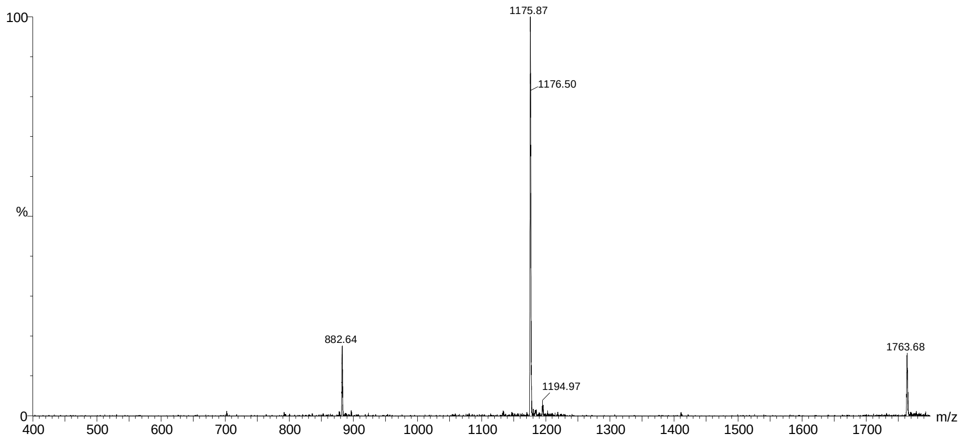

Table2: Peptide 35: upper panel: UPLC-UV Analysis on Waters Acquity BEH C18 column (2.1x 100 mm, 1.7 μm, 130 A), Waters TUV detector λ= 214 nm, solvent A= H2O, 0.1% TFA; B: CH3CN, 0.1% TFA; gradient of B 20%-20% (1')-60% (4'). Lower panel: ESI mass spectrometry on Waters SQ detector: MW theoretical= 3450.74; MW calculated = 3450.33

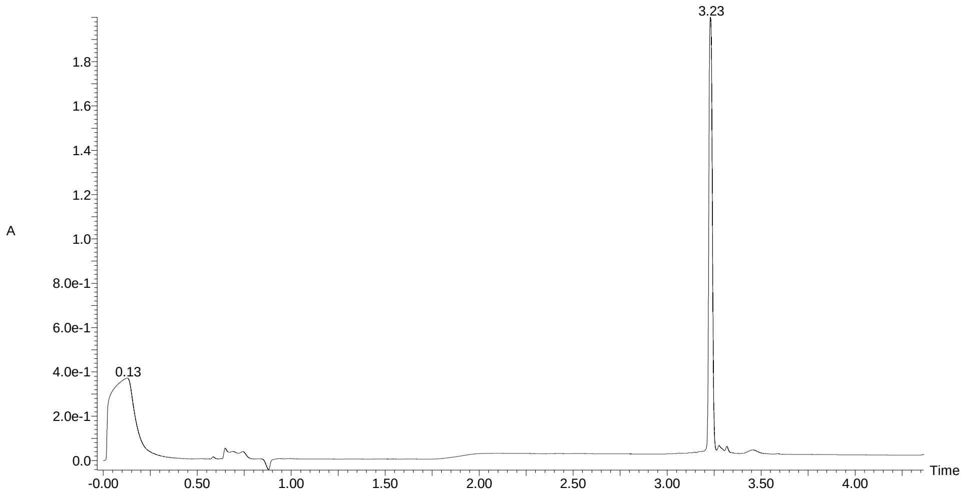

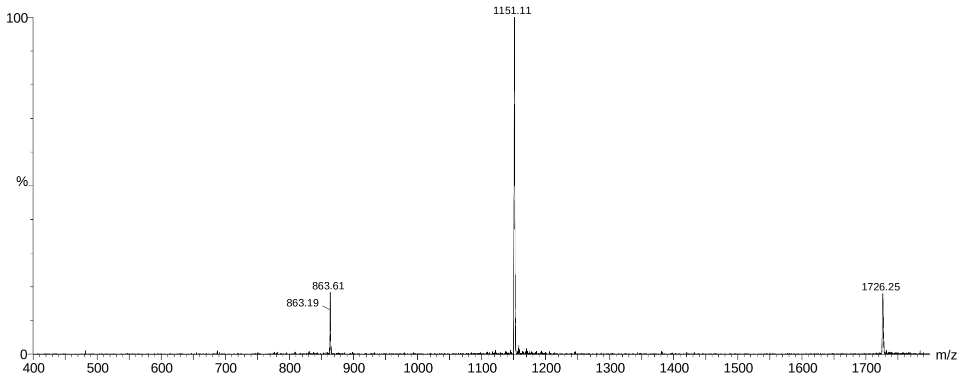

Table3: Peptide 36: upper panel: UPLC-UV Analysis on Waters Acquity BEH C4 column (2.1x 100 mm, 1.7 μm, 300 A), Waters TUV detector λ= 214 nm, solvent A= H2O, 0.1% TFA; B: CH3CN, 0.1% TFA; gradient of B 30%-30% (1')-70% (4')-90(1'). Lower panel: ESI mass spectrometry on Waters SQ detector: MW theoretical= 3934.43; MW calculated = 3934.14

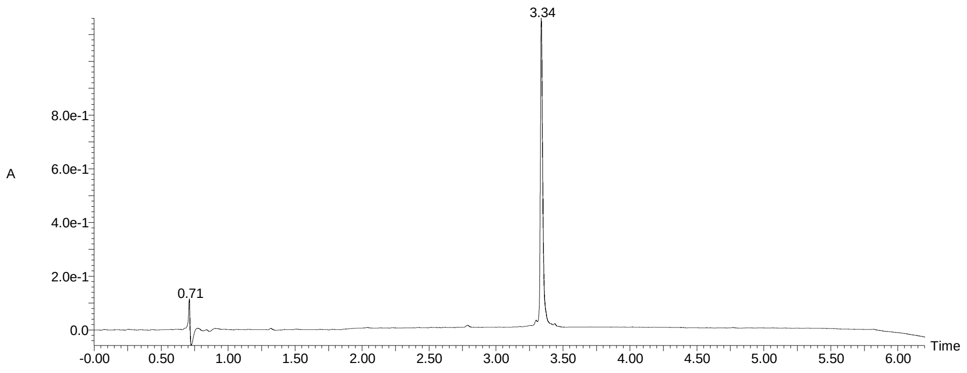

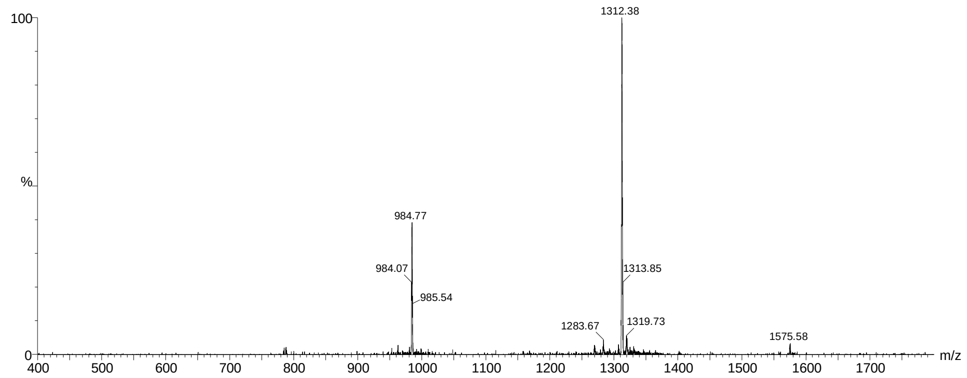

Table3: Peptide 37: upper panel: UPLC-UV Analysis on Waters Acquity BEH C4 column (2.1x 100 mm, 1.7 μm, 300 A), Waters TUV detector λ= 214 nm, solvent A= H2O, 0.1% TFA; B: CH3CN, 0.1% TFA; gradient of B 30%-30% (1')-70% (4')-90(1'). Lower panel: ESI mass spectrometry on Waters SQ detector: MW theoretical= 3919.52; MW calculated = 3920.1

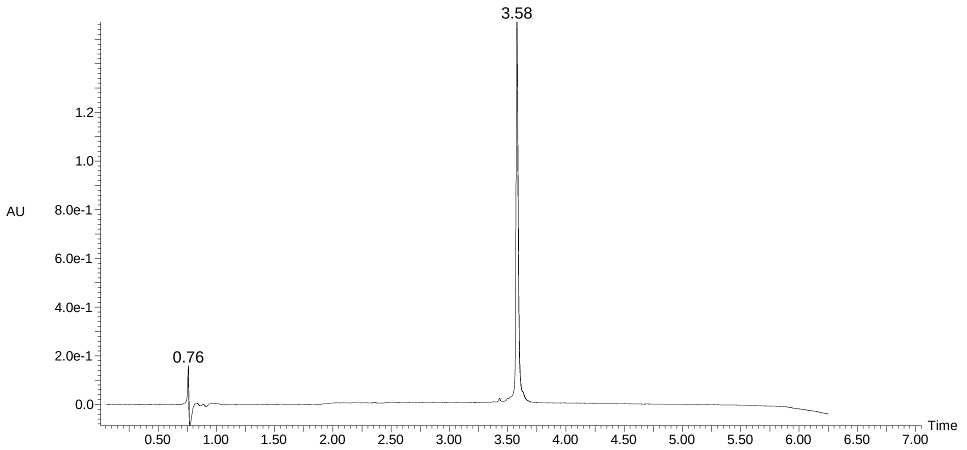

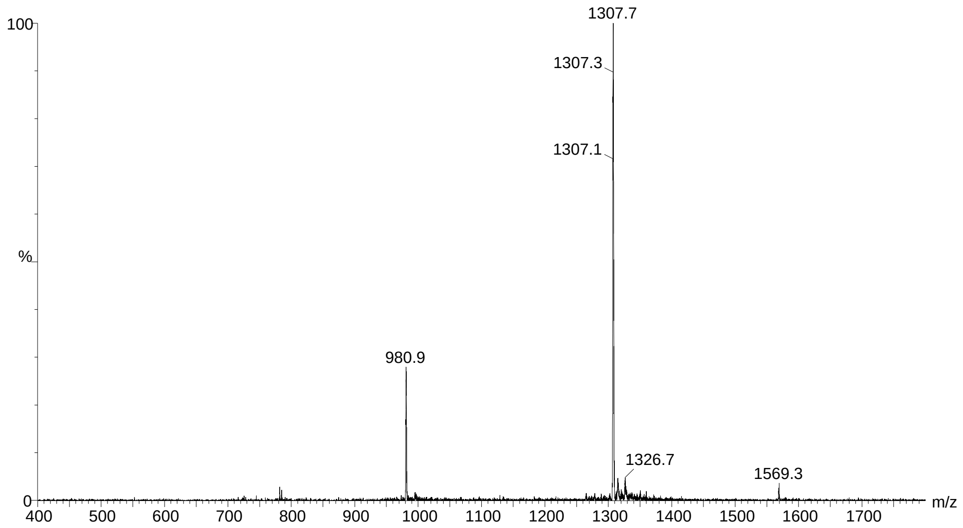

1307.7
1307.3
1307.1
980.9
1326.7
1569.3
100
%
0
400
500
600
700
800
900
1000
1100
1200
1300
1400
1500
1600
1700
m/z
